# Giant Berry‐phase‐Driven X‐Ray Beam Translations in Strain‐Engineered Semiconductor Crystals

**DOI:** 10.1002/adma.202513259

**Published:** 2025-10-30

**Authors:** Marco Felici, Giorgio Pettinari, Michela Fratini, Luisa Barba, Simone Birindelli, Gaetano Campi, Silvia Rubini, Tobias Schülli, Mario Capizzi, Antonio Polimeni

**Affiliations:** ^1^ Physics Department Sapienza University of Rome Rome 00185 Italy; ^2^ Institute for Photonics and Nanotechnologies (CNR‐IFN) National Research Council Rome 00133 Italy; ^3^ Institute of Nanotechnology (CNR‐Nanotec) National Research Council, c/o Physics Department Sapienza University of Rome Rome 00185 Italy; ^4^ Santa Lucia Foundation Rome 00179 Italy; ^5^ Institute of Crystallography (CNR‐IC) National Research Council Basovizza (Trieste) 34149 Italy; ^6^ Institute of Crystallography (CNR‐IC) National Research Council Monterotondo (Rome) 00015 Italy; ^7^ Istituto Officina dei Materiali, (CNR‐IOM) National Research Council Basovizza (Trieste) 34149 Italy; ^8^ ESRF ‐ The European Synchrotron Grenoble 38000 France

**Keywords:** berry‐phase translation effect, dilute nitride semiconductors, hydrogen doping, strain engineering, X‐ray photonics

## Abstract

The manipulation of light through its interactions with artificially structured media is a cornerstone of photonics. The rescaling of this concept to the X‐ray realm—which will enable us to control X‐ray light with the same precision routinely available in the visible/IR range—has so far been hindered by the inherent difficulty of realizing photonic structures with the sub‐nanometric resolution dictated by X‐ray wavelengths. A promising approach to this challenge is based on the so‐called Berry‐phase effect, the large beam translations undergone by X‐ray photons propagating in a deformed crystal, due to the simultaneous presence of Berry curvatures in real and reciprocal space. In this work, the controlled crystal distortions required to rein in this effect are obtained by pairing the lattice expansion observed upon H irradiation of GaAsN with a spatially selective hydrogenation technique. The macroscopic beam translations measured here are striking manifestations of the Berry curvatures associated with the sub‐nanometric lattice distortions induced by H incorporation. Through the comparison with a dedicated theoretical model, the individual translation branches observed in X‐ray transmission can be traced back to specific deformation features present within the samples, establishing a predictive framework for the control of X‐ray propagation in the fabricated structures.

## Graphical abstract

Due to the Berry‐phase effect, X‐rays propagating in deformed crystals undergo large translations, interesting for X‐ray optics applications. Here, the lattice expansion observed upon H irradiation of dilute‐nitride semiconductors is exploited to engineer the deformation landscape of selectively hydrogenated GaAsN epilayers. Beam translations >100 µm are subsequently observed and linked to specific deformation regions through the comparison with a dedicated model.

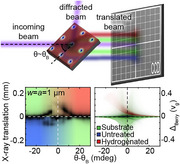

## 1 Introduction

The exploitation of the ability to control light through its interactions with matter is, arguably, both the core founding principle and the ultimate goal of photonics. In the last few decades, the boundaries of this field have been continuously pushed forward by making light interact with artificially structured systems, customizable within the (ever‐evolving) limits set by state‐of‐the‐art nanofabrication methods.^[^ ^1^ ^]^ Within this context, periodic photonic structures (PPSs)—such as photonic crystals,^[^ ^2^, ^3^ ^]^ distributed Bragg reflectors,^[^ ^4^, ^5^ ^]^ circular Bragg gratings,^[^ ^6^, ^7^ ^]^ plasmonic arrays,^[^ ^8^, ^9^ ^]^ and active photonic devices based on liquid crystals^[^ ^10^, ^11^ ^]^—have been particularly successful, giving rise to a wide range of applications. In essence, such structures aim at rescaling the diffractive phenomena^[^ ^12^ ^]^ naturally occurring in crystalline solids—due to the interaction of X‐ray photons with the periodic lattice, and characterized by wavelengths of the order of the lattice parameter (1–10 Å)—to the operating wavelength of interest, by defining periodic structures with the required spacing (100 nm–1 µm for devices working in the visible/near‐infrared interval).^[^ ^13^ ^]^ Moreover, the man‐made nature of PPSs entails the opportunity to deliberately disrupt the system's periodicity, thus introducing new functionalities. In photonic crystals, for example, high‐performance optical microcavities and waveguides can be easily realized by creating^[^ ^14^ ^]^—and subsequently optimizing^[^ ^15^, ^16^, ^17^, ^18^ ^]^—point and line defects in the photonic lattice.

**Figure 1:**
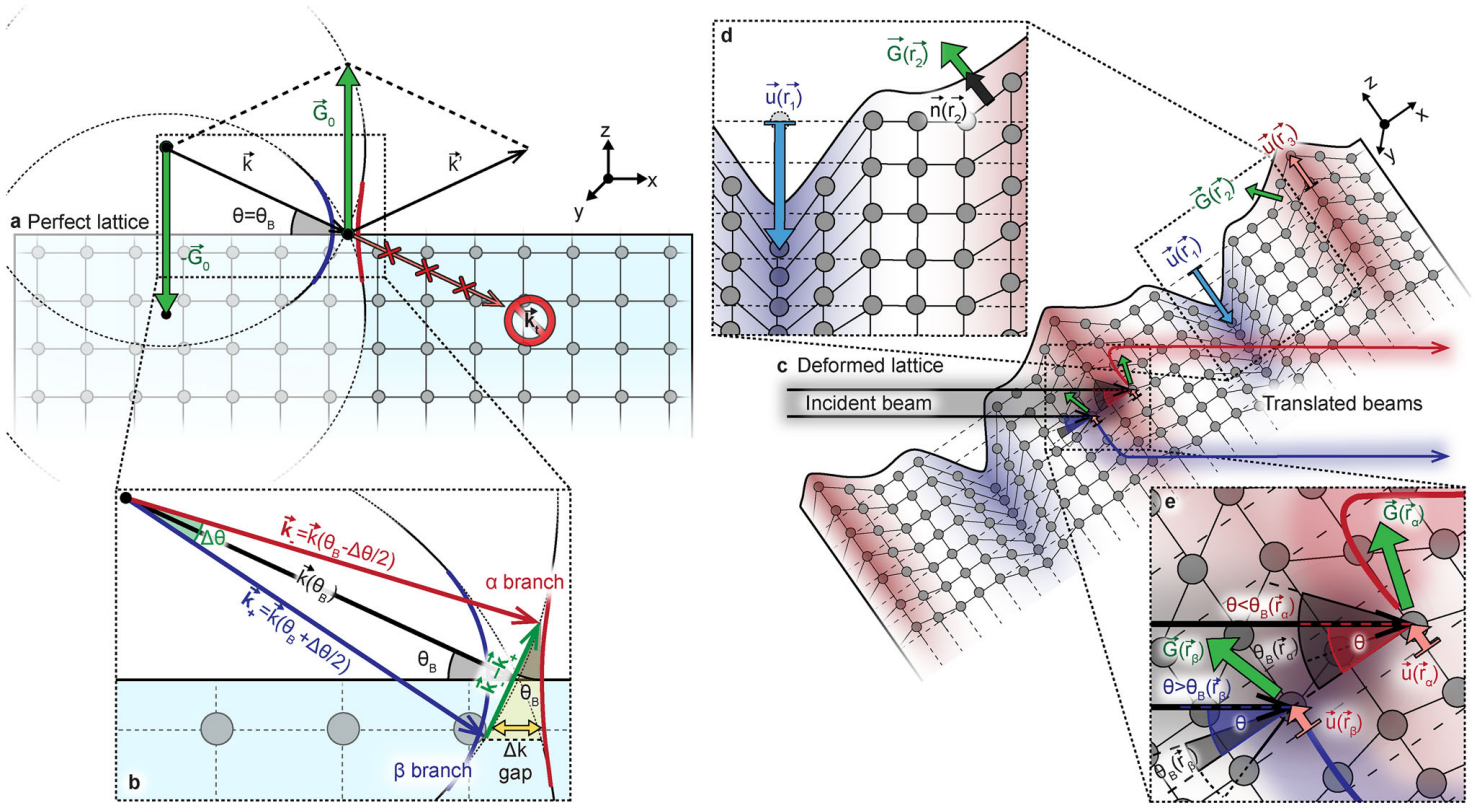
a) Schematic representation of an X‐ray beam impinging on a perfect crystal at the Bragg condition [${\vec{k}}^{\prime }-\vec{k}={\vec{G}}_{0}$, with $\;{\vec{G}}_{0}$ corresponding to the reciprocal lattice vector relative to the (004) reflection of undeformed GaAs]. Under this configuration—and as long as $\vec{k}$ falls within the gap characterizing the dispersion relation for X‐ray photons in the material, also sketched in the figure—the incident photons are fully diffracted, i.e., conventional light propagation cannot occur inside the crystal. This is graphically rendered by drawing the wavevector of the transmitted beam, $\vec{k_{t}}$, as a light‐red, crossed‐out arrow. b) Magnified view of the two branches of the dispersion relation for X‐ray photons in the crystal, respectively labeled as α (highlighted in red) and β (blue) and separated by a gap having size Δk. As discussed in the Experimental Section section, Δk is directly proportional to the angular Darwin width of the material, Δθ, also shown in the figure (see also Note S2, Supporting Information). c) Sketch of the Berry‐phase translation effect for X‐rays propagating in a deformed crystal. The regions characterized by an expanded (compressed) lattice are shaded in red (blue). The axes of the reference system (visible in the upper right corner of the figure) are parallel to the <100> crystallographic directions of the undeformed lattice. d) Zoom‐in of a highly deformed region of the crystal, highlighting the lattice properties that rule the spatial dependence of the Berry curvature tensor, $\Omega_{\vec{k}\vec{r}}$: the lattice deformation $\vec{u}\left(\vec{r}\right)$ and the reciprocal lattice vector $\vec{G}\left(\vec{r}\right)$ relevant to the experiments reported in this work (see main text). Also sketched is the $\hat{n}\left(\vec{r}\right)$ versor, perpendicular to the sample's surface and parallel to the $\vec{G}\left(\vec{r}\right)$ vector. e) Magnified view of the region in which the X‐ray wave packet and the lattice interact. In particular, we highlight two lattice locations, ${\vec{r}}_{\alpha }$ and ${\vec{r}}_{\beta }$, wherein nearly identical lattice deformations [$\vec{u}\left({\vec{r}}_{\alpha }\right)\approx \vec{u}\left({\vec{r}}_{\beta }\right)$] and values of $g_{r_{j}}\left(\vec{r}\right)$ (see Experimental Section) lead to diametrically opposite beam translations.

Over the last two decades, Kohmura, Sawada, and co‐workers^[^ ^19^, ^20^, ^21^, ^22^ ^]^ pioneered an innovative approach aimed at bringing the concepts behind PPSs back to the X‐ray realm. This approach is based on the very large translations undergone by an X‐ray beam propagating through a deformed crystal lattice. As postulated in a 2006 paper,^[^ ^19^ ^]^ which predicted this effect and developed a theoretical formalism to describe it, an X‐ray wave travelling through a deformed periodic medium strongly “feels” the presence of Berry curvatures^[^ ^23^ ^]^ in both real and reciprocal space.^[^ ^19^, ^20^, ^21^ ^]^ These curvatures, associated with the parametric dependence of the propagating electromagnetic wave packet on its position $\vec{r}$ and wavevector $\vec{k}$, are respectively due to the crystal deformation and to the lattice periodicity, with the latter leading to the opening of a band gap, Δk, in the dispersion relation of the propagating X‐rays. As sketched in **Figure** **1**, indeed, the photons impinging on a perfect crystal with an angle of incidence near (or at) the Bragg condition are fully diffracted by the lattice, i.e., they cannot propagate within the medium (hence, the emergence of a photonic gap; see Figure 1a,b). The presence of crystal deformations, while making photon propagation partially permitted, simultaneously triggers the so‐called Berry‐phase effect, whereby small (typically sub‐nanometer) lattice distortions are effectively transduced into large translations (i.e., lateral shifts) of the transmitted X‐ray beam (see Figure 1c–e). While we refer the reader to the Methods (and to the Supporting Information) for a detailed description of the processes leading up to the emergence of the Berry‐phase translation effect, here we note that i) the gap width, Δk, is a crucial parameter for the determination of the magnitude of this effect, as the maximal beam translation is inversely proportional to Δk; and that ii) in non‐monotonically deformed crystals—such as those investigated here or in ref. [21]—the sign of the translation (that is, the direction of the lateral shift) depends both on $\vec{k}$ (more specifically, on the angle of incidence of the impinging radiation) and on $\vec{r}$, the point of the lattice in which the X‐rays interact with the sample. As a result, different sample regions give rise to distinct translation branches, clearly visible in the transmission data presented in Section 2.2.

Returning to our summary of the key results underpinning the present study (see ref. [19, 20, 21, 22]), the first experimental demonstration of the Berry‐phase effect was reported in 2010, by measuring a ≈1 mm translation for an X‐ray beam propagating in a uniformly curved Si slab.^[^ ^20^ ^]^ Then, in 2013, ≈100 µm translations were observed for X‐rays transmitted through a Si crystal strained by the presence of Ge islands.^[^ ^21^ ^]^ In this latter case, two distinct translated beams were found to simultaneously emerge from the sample. This observation, coupled with the fact that the propagation direction of the translated beams remained parallel to that of the impinging wave packet,^[^ ^22^ ^]^ opened new prospects for the realization of innovative X‐ray optical components based on this effect. Particularly intriguing, in this context, is the possibility to fabricate X‐ray beam splitters and routers,^[^ ^20^, ^21^, ^22^ ^]^ potentially useful for applications in pump‐and‐probe experiments and in X‐ray interferometry,^[^ ^24^, ^25^, ^26^, ^27^ ^]^ as well as for beamline multiplexing at synchrotrons and X‐ray free‐electron lasers.^[^ ^28^ ^]^ Unlike conventional Bragg beam splitters,^[^ ^29^, ^30^ ^]^ Bonse–Hart crystals,^[^ ^24^ ^]^ or grating‐based interferometers^[^ ^25^, ^26^, ^27^, ^31^ ^]^—which are typically bulky, alignment‐sensitive, and/or relying on the fabrication of high‐aspect‐ratio, deeply etched components—the compact, monolithic devices based on the Berry‐phase effect would be able to generate two or more parallel, laterally displaced beams,^[^ ^20^, ^21^ ^]^ in principle enabling the on‐chip routing of X‐rays.

To fully reach its potential, however, this approach would require the availability of novel strain‐engineering protocols, allowing for the ad hoc tailoring of the lattice deformations encountered by the propagating beam and, thus, for the realization of optimized PPSs operating in the X‐ray range. What we propose here is an innovative method for achieving such arbitrary strain modulations, based on the spatially selective hydrogenation of dilute‐nitride semiconductors.^[^ ^32^ ^]^ As widely reported in the literature,^[^ ^33^ ^]^ indeed, the introduction of small percentages (1–2%) of nitrogen in III‐V compounds—such as GaAs and GaP—has disruptive effects on the properties of the host material. In particular, the anomalously large, N‐induced bandgap bowing—coupled to the reduction of the lattice parameter associated with N incorporation^[^ ^34^ ^]^—has attracted considerable attention, and should allow for the realization of, e.g., lattice‐matched InGaAsN/GaAs devices emitting in the 1.3–1.55 µm range of interest for telecommunications.^[^ ^35^, ^36^ ^]^ More recently, however, one of the main driving forces behind the investigation of the properties of dilute nitrides—particularly GaAsN,^[^ ^37^, ^38^, ^39^ ^]^ but also GaPN^[^ ^40^ ^]^ and InGaAsN^[^ ^32^, ^41^, ^42^ ^]^—has been represented by the discovery of the possibility to neutralize all N‐related effects by hydrogen irradiation. Following the formation of stable N‐H complexes (≈2 eV binding energies^[^ ^43^ ^]^), indeed, hydrogenation permanently modifies the electronic^[^ ^32^, ^40^, ^44^, ^45^ ^]^ and structural^[^ ^38^, ^42^, ^46^, ^47^ ^]^ properties of the host. This strikingly includes a full reversal of the N‐induced band‐gap reduction, which, paired with the possibility to modulate the H content in the material by selectively masking the sample surface before hydrogenation,^[^ ^48^ ^]^ allows for tailoring the in‐plane energy landscape of the sample at the nanometer scale,^[^ ^39^ ^]^ thereby creating arbitrary GaAsN/GaAsN:H hetero‐ and nanostructures (e.g., quantum dots^[^ ^49^, ^50^ ^]^). Within the framework of the present study, however, it is most interesting to note that the effects of H irradiation on the electronic properties of dilute nitrides are accompanied by similarly striking consequences at the structural level. In all these materials, indeed, the formation of N‐H complexes leads to a significant expansion of the unit cell, to the point that the lattice constant of, e.g., fully hydrogenated GaAsN actually exceeds that of pure GaAs.^[^ ^46^, ^51^, ^52^ ^]^ In epitaxially grown GaAsN/GaAs heterostructures, therefore, the character of the strain accumulated in the N‐containing layer can be tuned from tensile to compressive by gradually increasing the incorporated H dose.^[^ ^52^ ^]^ This suggests the possibility of exploiting spatially selective hydrogenation to obtain the controlled structural deformations required to harness the Berry‐phase translation effect. The steps required for the practical implementation of this approach are discussed in the next Section.

## 2 Results

### 2.1 Sample Fabrication and Computation of Structural Parameters

**Figure 2:**
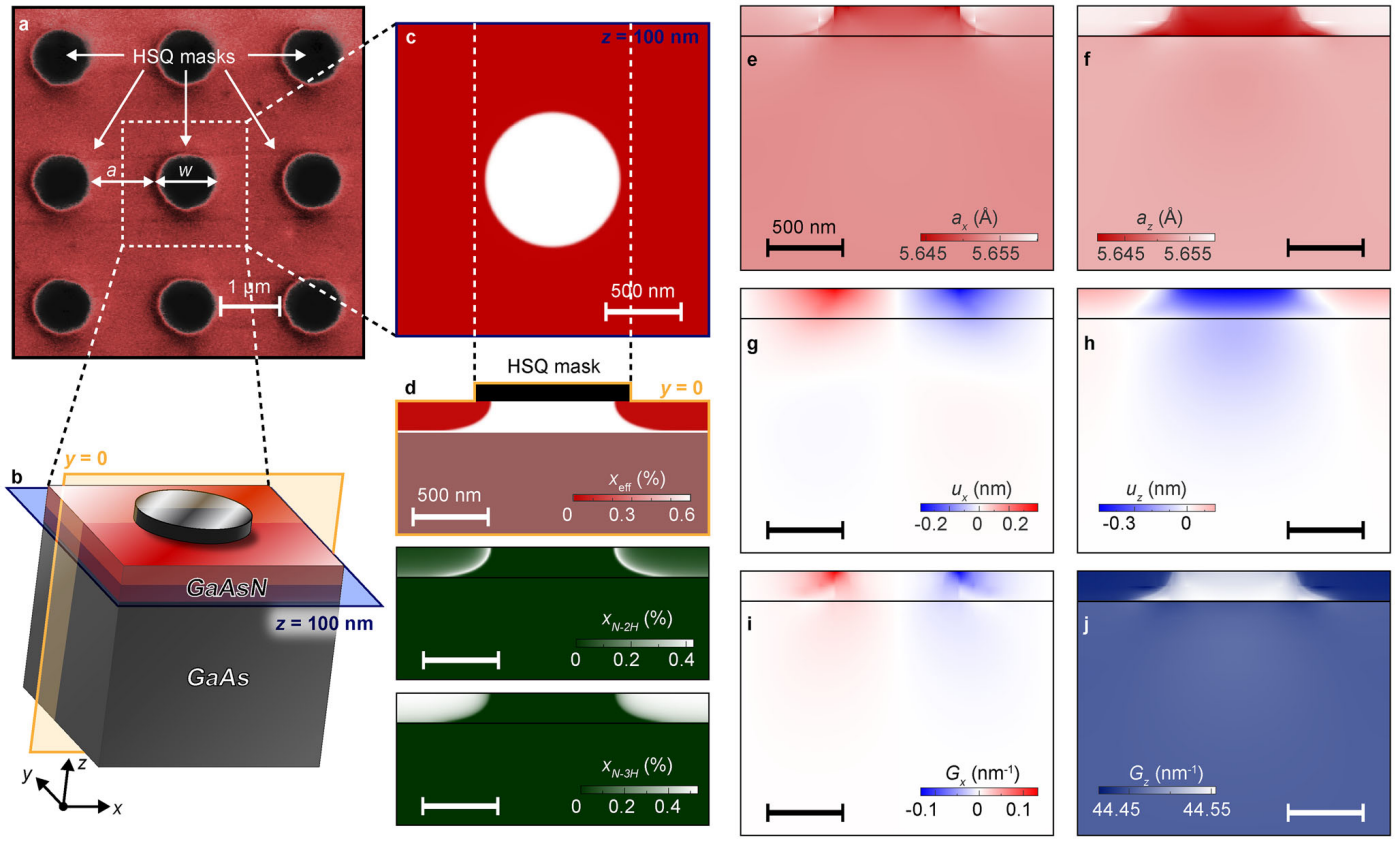
a) SEM image (in false colors) of an array of EBL‐defined, H‐opaque HSQ masks deposited on the sample surface before H irradiation. Both the mask diameter, *w*, and the edge‐to‐edge spacing, *a*, are equal to 1 µm in the displayed sample. b) 3D view of the 200‐nm‐thick GaAsN epilayer (deposited on GaAs by MBE; see Experimental Section) in the surroundings of a circular mask. The displayed region—also highlighted by a white‐dashed square in panel (a)—has a lateral size of *w*+*a*, and it corresponds to the computational domain employed for all the FEM calculations performed in this work (see Experimental Section and the following panels, which all refer to the *w* = *a* = 1 µm sample). c) Spatial distribution of the effective N concentration, *x_eff_*, in the *z* = 100 nm plane [highlighted in blue in panel (b)]. d) Top panel: same as (c) but for the *y* = 0 plane [highlighted in yellow in panel (b); all the computed spatial distributions displayed in the following panels refer to this plane]. Center and bottom panels: computed concentration maps of the N‐2H and N‐3H complexes (see main text and Experimental Section). e, f) Spatial distribution of *a_x_* (e) and *a_z_* (f), the lattice parameters in the *x* and *z* direction, respectively. g, h) Distribution of *u_x_* (g) and *u_z_* (h), the lattice deformations in the *x* and *z* direction. i, j) Spatial distribution of the reciprocal lattice vector relative to the (004) reflection of the deformed crystal. The components of the reciprocal lattice vector in the *x* and *z* directions, *G_x_* and *G_z_*, are respectively displayed in panels (i, j).

As detailed in the Experimental Section, the investigated samples (see **Figure** **2**) were fabricated via spatially selective H irradiation of a GaAsN/GaAs epilayer.^[^ ^50^ ^]^ Prior to hydrogenation, the sample's surface was covered with a square lattice of H‐opaque circular masks, having diameter *w* = 0.2, 0.5, or 1 µm and edge‐to‐edge spacing *a* = 1 µm (*w* = 1 µm for the scanning electron microscope (SEM) image in Figure 2a). As shown in Figure 2c,d, the anisotropic H distribution obtained with this method engenders a strong spatial modulation of *x_eff_*, the effective nitrogen concentration (i.e., the fraction of N atoms not bound to H) in the hydrogenated sample. Due to the very sharp diffusion profile of H in dilute nitrides (≈10–15 nm/decade at *T_H_* = 300 °C),^[^ ^39^ ^]^ the transition between fully hydrogenated and virtually H‐free regions is extremely abrupt, so that the periodic pattern defined by the H‐opaque masks is accurately transferred to the GaAsN epilayer. Given the sizeable lattice expansions associated with the formation of N‐H complexes (see Experimental Section, as well as ref. [46, 51]) the ability to induce periodic modulations in the H distribution provides a way to establish controlled, large‐scale deformations of the host lattice. The hydrogenation conditions were optimized to saturate the sample with N‐3H complexes (see Experimental Section and Figure 2d)—the stable species associated with the largest increase of the lattice parameter of the host^[^ ^51^ ^]^—thus obtaining a maximally deformed crystal.

As also discussed in ref. [53], the maps reported in Figure 2c,d were obtained by Finite‐Element Method (FEM) calculations (see Experimental Section). Notably, their availability is the starting point for an additional set of FEM calculations aimed at computing detailed maps of the lattice deformations created via the selective hydrogenation process. The results of these calculations are shown in Figure 2e–j, which displays the spatial distribution of the structural parameters most relevant to the present discussion. The *x* and *z* components of the lattice constant (*a_x_* and *a_z_*) and of the lattice deformation (*u_x_* and *u_z_*) are shown in Figure 2e–h, respectively. Figure 2i,j, on the other hand, displays the spatial distribution of *G_x_* and *G_z_*, the *x* and *z* components of the reciprocal lattice vector, $\vec{G}\left(\vec{r}\right)$, relative to the (004) reflection of the deformed crystal. As discussed in the Experimental Section, our FEM calculations were adjusted to account for the possibility that the H‐induced deformation of the GaAsN epilayer may trigger a distortion of the GaAs substrate in proximity of the GaAsN/GaAs interface, in a way similar to that reported in ref. [54] The results shown in Figure 2g,h, indeed, would seem to confirm the presence of a small but non‐negligible lattice deformation in the upper portion of the GaAs substrate, beneath the selectively hydrogenated GaAsN layer; in the *z* direction, for example, the maximal deformation of the substrate is ≈0.2 nm, to be compared with the ≈0.4 nm observed in GaAsN. As we will see in the following, correctly accounting for such substrate deformation is crucially important to obtain a satisfactory agreement between the experimentally measured Berry‐phase translation effect and its computational estimate.

**Figure 3:**
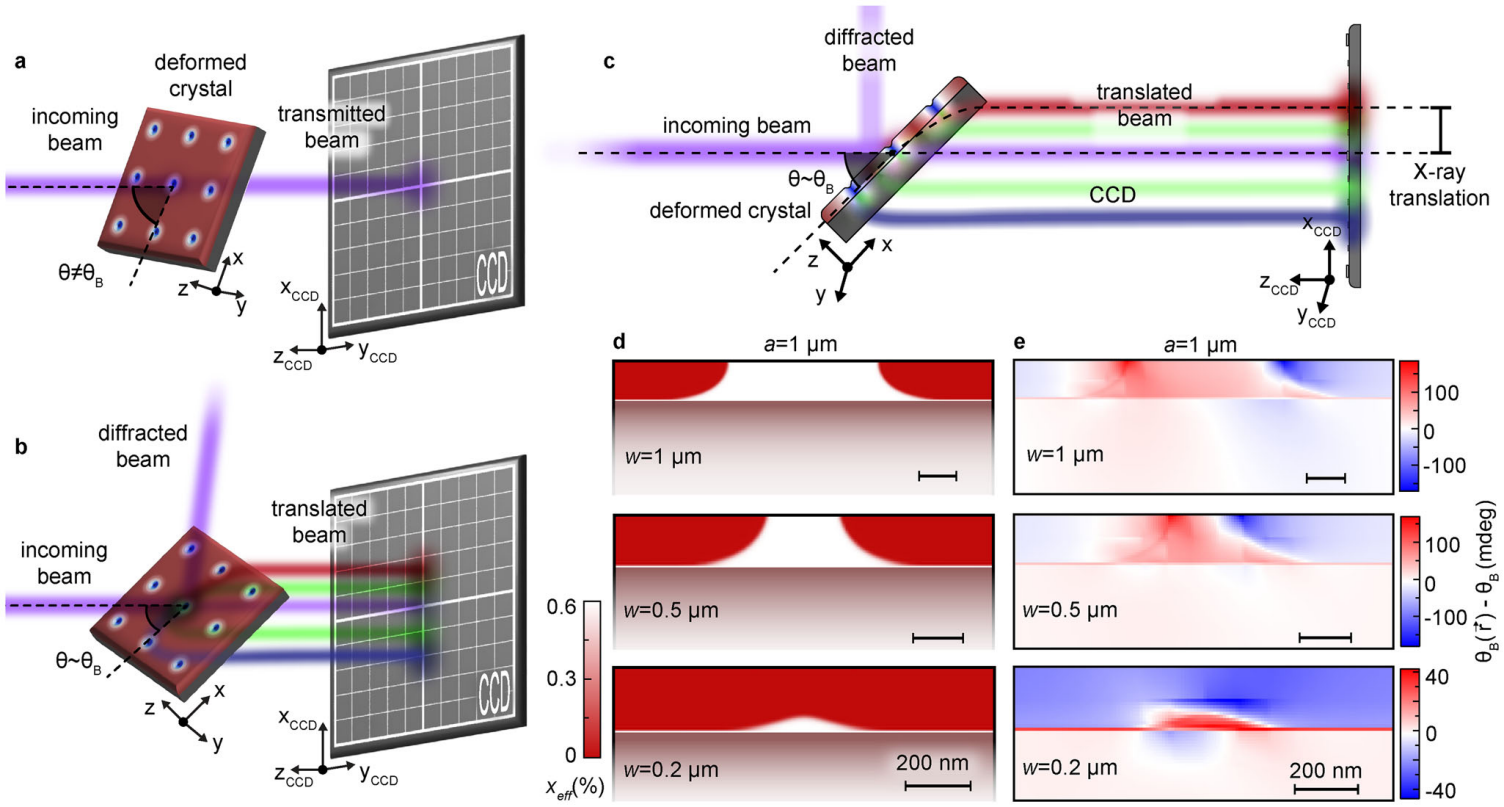
a,b) Sketch of the experimental configuration employed to measure the X‐rays transmitted by our selectively hydrogenated GaAsN samples. (a) For $\theta \neq \theta_{B}$, X‐rays is conventionally transmitted, and the beam impinging on the CCD detector placed behind the sample is collinear with the incoming beam. Here, *θ*_B_ is the Bragg angle corresponding to the (004) reflection of GaAs at the energy of the X‐rays employed here (10.33 keV). (b) For *θ* ∼ *θ*_B_, the Berry‐phase translation effect can be observed, with the appearance of two or more transmission branches corresponding to the interaction of the propagating X‐rays with different regions of the deformed sample (see main text). c) Side view of the experimental configuration shown in (b), highlighting how the translated beams are entirely contained in the plane defined by the incident X‐ray beam and by the *z* axis (see Note S3, Supporting Information). d) Spatial distributions of the effective N concentration (*x_eff_*) in the *y* = 0 plane (shown in yellow in Figure 2b), as obtained via FEM calculations of the H diffusion process in the masked GaAsN samples. From bottom to top, the displayed distributions refer to samples patterned with an array of circular masks having *a* = 1 µm and *w* = 0.2, 0.5, and 1 µm. e) Spatial distributions—computed for the same samples displayed in panel (d)—of the Bragg angle $\theta_{B}\left(\vec{r}\right)$, locally corresponding to the (004) reflection of the deformed lattice.

Clearly, the creation of a controlled lattice deformation in the hydrogenated GaAsN epilayer also implies that its reciprocal lattice vectors are no longer constant across the sample. Figure 2i,j summarizes the spatial dependence of the reciprocal lattice vector associated with the (004) reflection of the deformed crystal, $\vec{G}\left(\vec{r}\right)$. As discussed in Note S1 (Supporting Information), $\vec{G}\left(\vec{r}\right)=4\cdot \frac{2\pi }{a_{z}\left(\vec{r}\right)}\cdot \hat{n}\left(\vec{r}\right)$, where $\hat{n}\left(\vec{r}\right)$ is the versor normal to the surface defined by the distorted (001) lattice plane containing $\vec{r}$. As shown in Figure 2i,j, the lattice distortion associated with the combined presence of N and H in the sample has a twofold effect on $\vec{G}\left(\vec{r}\right)$: on the one hand, as it is obvious, its absolute value—$|\vec{G}\left(\vec{r}\right)|=4\cdot \frac{2\pi }{a_{z}\left(\vec{r}\right)}$—is deeply affected by the spatial dependence of $a_{z}\left(\vec{r}\right)$. This leads to the establishment of a pronounced positional dependence of the Bragg angle, $\theta_{B}\left(\vec{r}\right)$, which, in the context of this work, corresponds to the angle at which the Berry‐phase translation effect is maximal [see Equation (5), in the Experimental Section]. On the other hand, due to the small, deformation‐induced rotations of $\hat{n}\left(\vec{r}\right)$, in the distorted lattice $\vec{G}\left(\vec{r}\right)$ is no longer always parallel to *z*, i.e., there are significant portions of the sample in which $\vec{G}\left(\vec{r}\right)$ acquires a non‐zero component in the *xy* plane, $G_{xy}\left(\vec{r}\right)$ (see Figure 2i). Even though the absolute magnitude of $G_{xy}\left(\vec{r}\right)$ is rather small if compared to $G_{z}\left(\vec{r}\right)$—for example, $|\frac{G_{xy}\left(\vec{r}\right)}{G_{z}\left(\vec{r}\right)}|<5\cdot 10^{-3}$ for every point of the sample depicted in Figure 2—the tilt induced in the direction of $\vec{G}\left(\vec{r}\right)$ can be quite significant, triggering a correspondingly large change of $\theta_{B}\left(\vec{r}\right)$—up to 200 mdeg, for the data reported in Figure 2 (see also Panel e of **Figure** **3**, below). This is a positively massive angular variation within the framework of this work, as evidenced, e.g., by the fact that $\theta_{B}\left(\vec{r}\right)$ is expected to range between 25.094° and 25.181°—merely an 87 mdeg difference—due to the changes of $a_{z}\left(\vec{r}\right)$ displayed in Figure 2f.

### 2.2 Experimental Results

In order to characterize the influence of the artificial deformation landscape introduced in our patterned GaAsN:H/GaAsN/GaAs samples, the transmission of X‐ray photons through the crystal was monitored by scanning the angle *θ* between the incident beam and the sample surface, within a 60 mdeg range centered on the Bragg angle of GaAs, *θ*_B_ (as sketched in Figure 3a‐c; see Experimental Section for more details).

**Figure 4:**
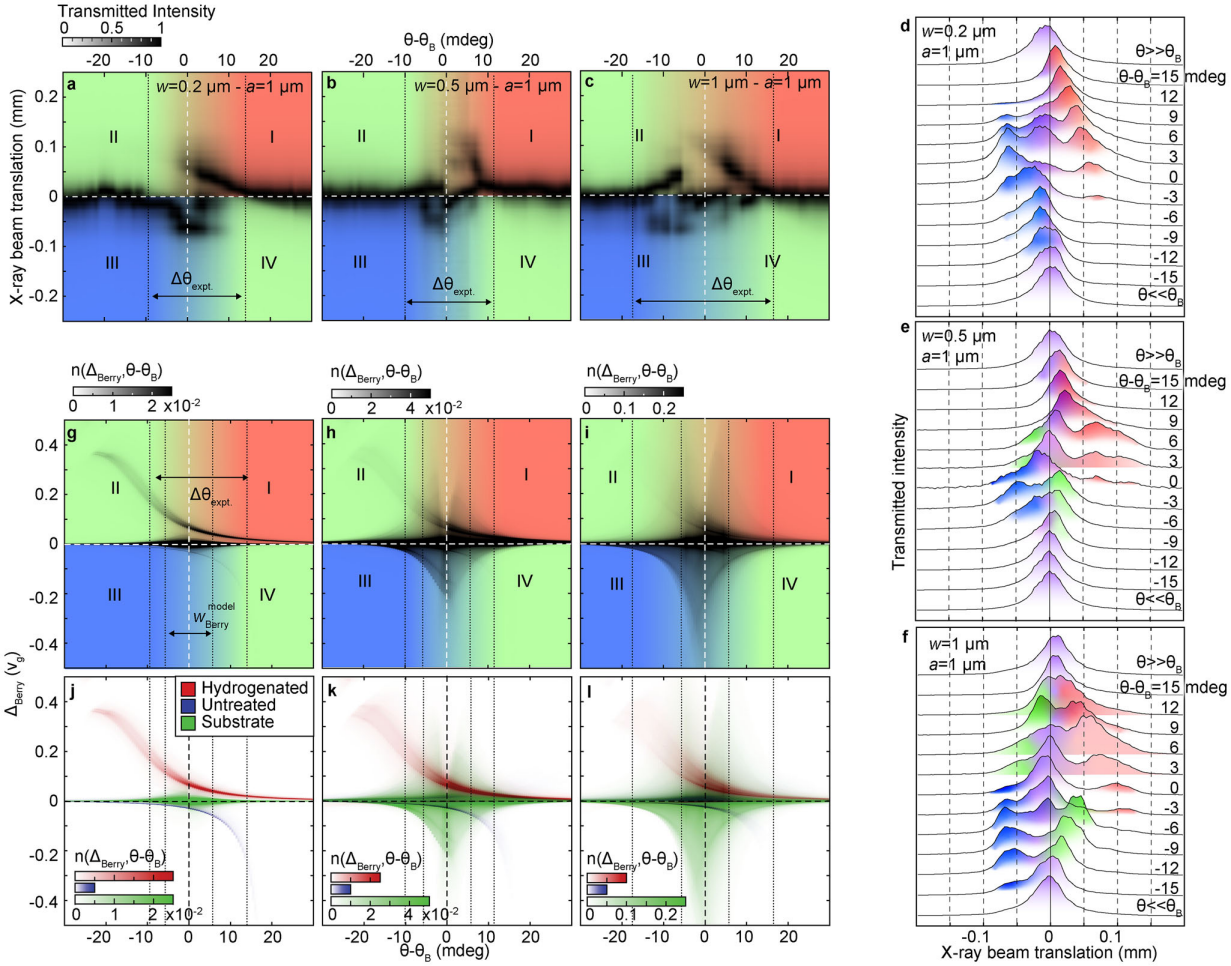
a–c) False‐color images of the intensity profile of the transmitted X‐ray photons—measured along the *x*_CCD_ axis of the detector, see Figure 3a–c—plotted as a function of $\theta -\theta_{B}$ (see main text and Experimental Section). The panels refer to the samples with *a* = 1 µm and *w* = 0.2 (a), 0.5 (b), and 1 µm (c), respectively. Each map is divided into four quadrants (see main text), shaded in red (quadrant I), blue (III), and green (II, IV). In each panel (and in the panels below), the low‐intensity windows measured in transmission (having a width equal to $\Delta \theta_{expt}$, see Note S4, Supporting Information) are identified by dotted lines. d–f) Stacked plots of the dependence on $\theta -\theta_{B}$ of the same intensity profiles displayed in (a–c), plotted as a function of the X‐ray translation with respect to the incident beam. The peaks falling in quadrants II‐IV in (a–c) are shaded in green, whereas those located in I and III are shaded in red and blue, respectively. g–i) False‐color plots of the $n(\Delta_{Berry},\theta -\theta_{B})$ distribution (see Note S3 and Figure S1, Supporting Information), computed for the samples with *a* = 1 µm and *w* = 0.2 µm (g), 0.5 µm (h), and 1 µm (i). For each sample, the color scales are artificially saturated to enhance the visibility of the finer features of $n(\Delta_{Berry},\theta -\theta_{B})$. The quadrants identified in (a–c) are also displayed here, with the same color scheme. For reference, in each panel (and in the panels below) we highlight (again with dotted lines) the region centered on $\theta =\theta_{B}$ and corresponding to $w_{Berry}^{model}$ = 11 mdeg, the angular width of the $f_{k_{i}}\left(\vec{k},\vec{G}\left(\vec{r}\right)\right)$ function [see Equation (3)] that results from using $\frac{\Delta k}{k}$ = $5\cdot 10^{-5}$ in our calculations (see Note S2 and Panel a of Figure S2, Supporting Information). j–l) Same as (g–i), save for the fact that the contributions to $n(\Delta_{Berry},\theta -\theta_{B})$ of the different sample regions identified in the inset of Figure S1a (Supporting Information) are shown in a dedicated color scale: red and blue for the hydrogenated and untreated regions, respectively, and green for the GaAs substrate. The color scales are individually saturated to maximize the visibility of each trace.

The trace plots presented in Panels a and c of **Figure** **4** display the intensity of the transmitted X‐rays as a function of the sample orientation—measured as $\theta -\theta_{B}$—and of the X‐ray beam translation with respect to conventionally transmitted photons (according to the notation introduced in Figure 3a‐c, the X‐ray translations were measured along the *x*_CCD_ direction; see Experimental Section). In order to better visualize the evolution of the transmission patterns of the X‐rays propagating through our samples, the plots displayed in Figure 4a‐c are divided into four quadrants, labeled from I to IV. If the Roman numeral “I” is assigned to the quadrant corresponding to positive values of both $\theta -\theta_{B}$ and the X‐ray translation, quadrants II to IV can be defined, moving counterclockwise, as those with $\theta -\theta_{B}<0$ and positive X‐ray translation (II), with negative values of both $\theta -\theta_{B}$ and the X‐ray translation (III), and with $\theta -\theta_{B}>0$ and negative X‐ray translation (IV). By comparing the traces shown in Figure 4a–c, corresponding to the samples with *w* = 0.2 µm (Figure 4a), 0.5 µm (Figure 4b), and 1 µm (Figure 4c), it is easy to notice that the traces falling within quadrants I and III—characterized by X‐ray translations having the same sign as their corresponding values of $\theta -\theta_{B}$—are present for all samples. This is also apparent from the stacked plots shown in Figure 4d–f, displaying, for increasing values of $\theta -\theta_{B}$, the beam profiles as a function of the X‐ray translation. The evolution of the transmission pattern with increasing *w* is most apparent in quadrants II and IV, wherein one can clearly see, for the samples with *w* = 0.5 and 1 µm, the progressive appearance of traces of a different kind, associated with X‐ray translations with a sign opposite to that of $\theta -\theta_{B}$.

Based on the arguments made above and in the Experimental Section (see Figure 1c–e, in particular), the distinct traces that can be identified in the transmission data most likely stem from the interaction of the propagating X‐ray photons with different regions of the crystal, characterized by well‐defined deformation landscapes. In Note S3 (Supporting Information), we discuss how to establish a correspondence between individual transmission branches and specific sections of our patterned samples, by pairing the structural information obtained from the FEM calculations described in the previous Subsection with the formalism introduced in the Experimental Section. As detailed in Note S3 (Supporting Information), the accomplishment of this goal goes through the definition of two quantities: $\Delta_{Berry}\left(\vec{k},\vec{r}\right)$ and $n(\Delta_{Berry},\theta )$. On the one hand, $\Delta_{Berry}\left(\vec{k},\vec{r}\right)$ is the relative change—with respect to a straight propagation—of the *z* component of the equation of motion for an X‐ray wave packet with wavevector $\vec{k}$, travelling through point $\vec{r}$ of a deformed medium. Integrated along the entirety of the trajectory covered by the X‐ray photons inside the material, the values taken by $\Delta_{Berry}\left(\vec{k},\vec{r}\right)$ yield the experimentally measured beam translation. On the other hand, $n(\Delta_{Berry},\theta )$ is the dependence on *θ* of the distribution of the values taken by $\Delta_{Berry}$ within our system. As we will see in the following Section, $n(\Delta_{Berry},\theta )$ can be directly—albeit qualitatively—compared with our experimental results, greatly aiding our interpretation of the latter. In particular, the trends observed in the measured X‐ray translations with increasing *w* (see Figure 4a–f) can be almost entirely explained in terms of the growing importance of the contribution of the deformations of the GaAs substrate to $n(\Delta_{Berry},\theta )$ (see Figure S1d,h, Supporting Information).

### 2.3 Comparison Between Theory and Experiments

Figure 4g–i displays the $n(\Delta_{Berry},\theta )$ distributions of the samples with *w* = 0.2, 0.5, and 1 µm, computed as described in Note S3 (Supporting Information) (see also Figure S1, Supporting Information). Remarkably, the evolution of $n(\Delta_{Berry},\theta )$ with increasing *w* reproduces quite well the trends observed in the experimental data. As shown in Figure 4a, indeed, the sample with *w* = 0.2 µm shows a simple anti‐crossing behavior, with the transmitted X‐rays undergoing a positive (negative) ≈100 µm translation for $\theta >\theta_{B}$ ($\theta <\theta_{B}$). The same anti‐crossing is displayed by the computed $n(\Delta_{Berry},\theta )$ distribution (see Figure 4g), featuring traces characterized by a maximal $|\Delta_{Berry}|$ of the order of ≈$0.5\cdot v_{g}$ (where $v_{g}$ is the group velocity of the X‐ray wave packet in a perfect crystal). In Figure 4j, the separate contributions of the different regions of the *w* = 0.2 µm sample to $n(\Delta_{Berry},\theta )$ are plotted together, in different color scales. It is interesting to note that the trace characterized by a positive value of $\Delta_{Berry}$ (in red in the figure) originates from the hydrogenated portion of the sample, whereas the blue trace, associated with negative $\Delta_{Berry}$, stems from the H‐free region. The contribution of the substrate—displayed in green—is mostly associated with low values of $\Delta_{Berry}$, consistent with the negligible substrate distortion engendered in the *w* = 0.2 µm sample by the selective hydrogenation procedure.

The picture changes for the samples with *w* = 0.5 and 1 µm, for which additional features, associated with beam translations having a sign opposite to that of $\theta -\theta_{B}$, progressively appear in the transmitted signal (see Figure 4b,c), making the total pattern visibly more symmetric with respect to *θ*_B_. Once again, this trend is qualitatively reproduced by the computed $n(\Delta_{Berry},\theta )$ distributions, which clearly become more symmetrical with increasing *w*, as displayed in Figure 4h,i. The analysis of the contribution of the different sample regions to $n(\Delta_{Berry},\theta )$—shown in Figure 4k,l for *w* = 0.5 and 1 µm—clearly demonstrates that the origin of the observed behavior stems from the progressive emergence of a series of additional traces, symmetric with respect to *θ*_B_ and associated with the GaAs substrate. With increasing *w*, these traces gradually overtake those associated with the H‐free and hydrogenated sections of the GaAsN epilayer, leading to the observed evolution of $n(\Delta_{Berry},\theta )$ and, thus, of the measured transmission patterns.

## 3 Conclusion

In conclusion, we demonstrated that the large translations experienced by the X‐ray photons propagating through a deformed crystal—due to the interplay between the real‐ and reciprocal‐space Berry curvatures present in these systems—can be triggered by purposedly altering the deformation landscape of an epitaxially grown GaAsN/GaAs epilayer via spatially selective hydrogen irradiation. More importantly, our ability to lithographically control the amount of H locally incorporated in each point of the GaAsN lattice can be exploited to fine‐tune the nuances of the experimentally measured transmission patterns by carefully crafting the deformation profile in the material. The possibility to enhance or depress the importance of the translations associated with different sample regions—the H‐free features and the fully hydrogenated barriers in the GaAsN epilayer, as well as the GaAs substrate—leads to the ability to activate, or deactivate, specific branches in the transmission patterns. These results open new, enticing prospects for the realization of tailor‐made X‐ray photonic structures and optical components.

In order to optimize the properties of these novel devices, in the follow‐up to this work, we plan to continue our exploration of the dependence of the X‐ray translations on the geometry of the deformation pattern, by, e.g., fabricating wire ensembles, by further varying *w* and *a* of the dot array, and by probing the properties of non‐periodic (random) arrays. In addition, we plan to probe in greater depth the role played by the properties of the GaAsN epilayer, i.e., the N concentration and the layer thickness, in the determination of the experimentally measured beam translations.

Finally, we would like to note that while our spatially selective hydrogenation technique allows for the definition of virtually arbitrary deformation landscapes—thus being uniquely suited for the realization of optical devices based on the Berry‐phase translation effect—it does so statically, i.e., the induced lattice deformation is basically fixed by the lithographic pattern and by the hydrogenation parameters. Although post‐fabrication H removal is possible, either globally (by thermal annealing^[^ ^55^ ^]^) or locally (e.g., via near‐field laser illumination^[^ ^56^ ^]^), these procedures do not provide a practical route to the dynamic tuning of the deformation profile. In this respect, surface acoustic waves, SAWs,^[^ ^57^, ^58^ ^]^ represent a potentially very powerful tool for triggering and/or modulating the Berry‐phase translation effect at a fast rate and over a virtually unlimited number of cycles. As a next step, we will thus be testing this approach in GaAs, wherein SAW‐induced lattice deformations of ≈1 nm have been reported.^[^ ^57^ ^]^ These values are consistent with those reported here and, e.g., in ref. [21] and are therefore sufficient to give rise to sizeable X‐ray translations. We will then move to selectively hydrogenated GaAsN, where the pairing of static and dynamic deformations will enable the implementation of even more complex strain‐engineering protocols. In parallel, we also see promising opportunities for investigating the Berry‐phase effect in 2D materials, whose inherent flexibility and ability to withstand very large lattice distortions^[^ ^59^ ^]^ have given rise to a plethora of strain‐engineering methods^[^ ^60^ ^]^—both static^[^ ^61^ ^]^ and dynamic,^[^ ^62^ ^]^ including SAW‐based approaches.^[^ ^63^ ^]^ Ultimately, these additional investigations could pave the way to the development of reconfigurable X‐ray optical components based on the Berry‐phase effect, featuring tunable beam translations that can be modulated and switched on and off in real time.

## 4 Experimental Section

### Theoretical Background

The authors of ref. [19, 20, 21] investigated the propagation of an X‐ray wave packet in a deformed medium, basing their analysis on a formalism inspired by the one developed in ref. [64] As defined in ref. [21], the *i*‐th component (*i* = *x,y,z*) of the equation of motion for the center position $\vec{r}$ of an X‐ray wave packet propagating in the presence of a crystal deformation can be written as:

(1) $$\frac{dr_{i}}{dt}=v_{g}^{i}+\sum_{j=x,y,z}\frac{dr_{j}}{dt}\Omega_{k_{i}r_{j}}$$

where $v_{g}^{i}$ is the *i*‐th component of the group velocity of the wave packet in a perfect crystal. The key parameter in Equation (1) is the so‐called Berry curvature tensor, $\Omega_{\vec{k}\vec{r}}$, which ultimately determines the deviation of the propagating wave packet from a straight trajectory. According to ref. [21], the elements of the $\Omega_{\vec{k}\vec{r}}$ tensor take the form:

(2) $$\Omega_{k_{i}r_{j}}=f_{k_{i}}\left(\vec{k}\left(\theta \right)\right)\cdot g_{r_{j}}\left(\vec{r}\right)$$

with;

(3) $$f_{k_{i}}\left(\vec{k}\left(\theta \right)\right)=\pm \frac{\frac{{\left(\vec{k}\left(\theta \right)+\vec{G}\right)}_{i}}{\left|\vec{k}\left(\theta \right)+\vec{G}\right|}-\frac{k_{i}\left(\theta \right)}{\left|\vec{k}\left(\theta \right)\right|}}{2\cdot {\left[{\left(\Delta k\right)}^{2}+\frac{1}{4}{\left(\left|\vec{k}\left(\theta \right)\right|-\left|\vec{k}\left(\theta \right)+\vec{G}\right|\right)}^{2}\right]}^{3/2}}\cdot {\left(\Delta k\right)}^{2}$$

and;

(4) $$g_{r_{j}}\left(\vec{r}\right)=\frac{\partial \left[\vec{G}\cdot \vec{u}\left(\vec{r}\right)\right]}{\partial r_{j}}$$

where $\vec{G}$ and $\vec{u}\left(\vec{r}\right)$ are the reciprocal lattice vector of interest [which, for the present work, is the one relative to the (004) reflection of the crystal, see above and Figure 1] and the local lattice displacement, respectively. As anticipated in the Introduction, a non‐zero $\Omega_{\vec{k}\vec{r}}$ (i.e., the emergence of a finite Berry‐phase translation) directly results from the interplay between the real‐space crystal deformation—as clearly evidenced by the explicit dependence of $g_{r_{j}}\left(\vec{r}\right)$ on $\vec{u}\left(\vec{r}\right)$—and the system's periodicity, which leads to the emergence of a finite‐sized gap—having width Δk—in the dispersion relation of the X‐ray photons propagating in the crystal. Such a gap is centered on the wavevector $\vec{k}$ complying with the Bragg condition—${\vec{k}}^{\prime }-\vec{k}=\vec{G}$, where $\vec{k}$ and ${\vec{k}}^{\prime }$ are relative to the incident and diffracted beams, respectively (see Figure 1a,b). Its opening is directly associated with the fact that the X‐rays impinging on the surface of a perfect crystal at an angle fulfilling the Bragg condition (*θ_B_*, the so‐called Bragg angle) are fully diffracted by the lattice, so that photons whose wavevector falls within the gap cannot propagate inside the material. Propagation in a deformed crystal, while no longer strictly forbidden, is deeply affected by the presence of the gap, whose width Δk is, clearly, a crucial parameter for the evolution of $f_{k_{i}}\left(\vec{k}\right)$ (and, thus, of the $\Omega_{\vec{k}\vec{r}}$ tensor) with $\vec{k}$ (see Equation (3)).

The geometric configuration displayed in Figure 1a—aside from respecting the Bragg condition, with reciprocal lattice vector, ${\vec{G}}_{0}$, corresponding to the (004) reflection of undeformed GaAs—represents a natural starting point for the X‐ray transmission measurements presented in this work. In this spatial configuration, the wavevector $\vec{k}$—hence labeled as $\vec{k}\left(\theta \right)$ in Equations (2) and (3)—can be varied by rotating the crystal in the *xz* plane, scanning the angle *θ* between the incident X‐ray beam and the sample surface. It is interesting to note that $f_{k_{i}}\left(\vec{k}\left(\theta \right)\right)$ changes sign as *θ* is swept across *θ_B_* [the ± sign in Equation (3) is, indeed, positive for $\theta <\theta_{B}$, while it is negative for $\theta >\theta_{B}$]. This leads to the emergence of two propagation branches, labeled as α and β in ref. [21] and in Figure 1b; clearly, these two branches correspond to the two bands that form because of the periodicity‐induced bandgap opening in the X‐ray dispersion relation, see above.

In addition, if $\vec{G}$ is parallel to, e.g., the *z* axis (as in the experimental configuration employed in this work, see Figure 1), from Equation (3) it can be inferred that:

(5) $$f_{k_{x},k_{y}}\left(\vec{k}\left(\theta \right)\right)\equiv 0\;\;\;\;\forall \theta \;\left|f_{k_{z}}\left(\vec{k}\left(\theta \right)\right)\right|\underset{\theta \to \theta_{B}}{\to }\frac{\sin\left(\theta_{B}\right)}{\Delta k}$$

This obviously entails that i) $f_{k_{i}}\left(\vec{k}\left(\theta \right)\right)$ is nonzero only for *k_i_* = *k_z_* (or, in general, in the direction parallel to $\vec{G}$), and that ii) the value of $|f_{k_{z}}\left(\vec{k}\left(\theta \right)\right)|$ reaches its maximum for $\theta =\theta_{B}$. Such a maximal value is inversely proportional to Δk and, ultimately, it determines the magnitude of the Berry‐phase translation effect for propagating X‐rays. Given that, as reported in ref. [19, 20, 21] Δk is vanishingly small (the $\frac{\Delta k}{k}$ ratio is typically of the order of 10^−5^–10^−6^ for hard X‐rays, with photon energy ≥5‐10 keV), $f_{k_{i}}\left(\vec{k}\left(\theta \right)\right)$ effectively serves as a $\vec{k}$‐dependent amplification factor for the $g_{r_{j}}\left(\vec{r}\right)$ term, transducing the—generally sub‐nm—lattice deformation into a beam translation that, for a wave packet impinging on the crystal at the Bragg condition, can be of the order of 1 mm, as reported in ref. [20] It is worth noting here that the origin of the small value of Δk lies in its direct relationship with a quantity of great importance for X‐ray diffraction: the intrinsic Darwin width of the system,^[^ ^12^ ^]^ Δθ. In Note S2 (Supporting Information) (see also Figure 1b), it is indeed demonstrated that:

(6) $$\frac{\Delta k}{k}∼\sin\left(\theta_{B}\right)\cdot \Delta \theta$$

Given that, for conditions similar to those encountered in our experiments, Δθ is known to be of the order of ≈10^−5^ rad (see, e.g., ref. [65]), this equation yields values of $\frac{\Delta k}{k}$ in perfect agreement with those reported in ref. [19, 20, 21].

After analyzing the $f_{k_{i}}\left(\vec{k}\right)$ factor, which univocally defines the dependence of the $\Omega_{\vec{k}\vec{r}}$ tensor on the wavevector $\vec{k}$ (see Equation (3))—and, thus, on the angle *θ* between the incident X‐ray beam and the sample surface—the attention is now shifted to the equally important issue of how $\Omega_{\vec{k}\vec{r}}$ depends on the real‐space coordinate, $\vec{r}$. According to Equations (2) and (4), the dependence of $\Omega_{\vec{k}\vec{r}}$ on $\vec{r}$ should be fully described by the $g_{r_{j}}\left(\vec{r}\right)$ term, which is in turn equal to the partial derivative (along the *r_j_* direction) of the scalar product between the reciprocal lattice vector, $\vec{G}$, and the lattice deformation, $\vec{u}\left(\vec{r}\right)$. While a single, constant $\vec{G}$ is sufficient to reproduce the Berry‐phase effect observed in the uniformly bent Si slab investigated in ref. [20] for the deformation studied in ref. [21] induced by the presence of Ge islands in a Si crystal, it is necessary to introduce two vectors, ${\vec{G}}_{1}$ and ${\vec{G}}_{2}$, to account for the different curvatures of the deformed surface. For the GaAsN:H/GaAsN/GaAs samples investigated in this work, however, the role played by the complex deformation patterns introduced in the crystal via spatially selective hydrogenation can only be properly taken into account by including the full spatial dependence of the reciprocal lattice vector—henceforth labeled as $\vec{G}\left(\vec{r}\right)$ in the model.

While it is trivial to observe that the inclusion of a position‐dependent $\vec{G}\left(\vec{r}\right)$ has significant effects on the $g_{r_{j}}\left(\vec{r}\right)$ term—which is no longer dominated by the evolution of $\vec{u}\left(\vec{r}\right)$ with $\vec{r}$, see Equation (4)—the $\Omega_{\vec{k}\vec{r}}$ tensor is also affected in a slightly less obvious, yet possibly much more significant fashion. As detailed in Note S2 (Supporting Information) (see also the sketches displayed in Figure 1c–e), in a deformed crystal, both the absolute value and the direction of $\vec{G}\left(\vec{r}\right)$ change as a function of position; as a result, the Bragg condition is also contingent on the real‐space coordinate. Given that this condition basically defines the $\vec{k}$‐vector for which $|f_{k_{i}}\left(\vec{k}\right)|$ reaches its maximum and $f_{k_{i}}\left(\vec{k}\right)$ changes sign [see Equations (3) and (5)], this obviously entails that $f_{k_{i}}\left(\vec{k}\right)$ is also position‐dependent, i.e., it is more accurate to write it as $f_{k_{i}}\left(\vec{k},\vec{G}\left(\vec{r}\right)\right)$. As previously anticipated, this additional effect of the inclusion of the spatial dependence of $\vec{G}\left(\vec{r}\right)$ on $\Omega_{\vec{k}\vec{r}}$ can lead to situations similar to that depicted in Figure 1e, which highlights two lattice locations, ${\vec{r}}_{\alpha }$ and ${\vec{r}}_{\beta }$, wherein nearly identical lattice deformations [$\vec{u}\left({\vec{r}}_{\alpha }\right)\approx \vec{u}\left({\vec{r}}_{\beta }\right)$] lead to diametrically opposite beam translations. Due to the different Bragg angles associated with the position‐dependent reciprocal lattice vectors, $\vec{G}\left({\vec{r}}_{\alpha }\right)$ and $\vec{G}\left({\vec{r}}_{\beta }\right)$, as well as to the different angles formed by the incident beam with the locally distorted crystal surface, the propagating photons fall either in the α branch or in the β branch of the photon dispersion relation (see Figure 1b), leading to the emergence of two separate translated beams. For the geometry sketched in Figure 1e, it is fair to assume that $g_{r_{j}}\left({\vec{r}}_{\alpha }\right)\approx g_{r_{j}}\left({\vec{r}}_{\beta }\right)$, so that the dramatically different behavior of two photons interacting with the lattice in ${\vec{r}}_{\alpha }$ and ${\vec{r}}_{\beta }$ (while having the same wavevector, $\vec{k}$) can be mainly ascribed to the dependence of $\vec{G}\left(\vec{r}\right)$—and, thus, of $f_{k_{i}}\left(\vec{k},\vec{G}\left(\vec{r}\right)\right)$—on the real‐space coordinate.

Of course, the situation sketched in Figure 1c–e is highly idealized; in real samples—such as the ones investigated here—the point‐by‐point dependence of the Berry curvature tensor $\Omega_{k_{i}r_{j}}\left(\vec{k},\vec{r}\right)=f_{k_{i}}\left(\vec{k},\vec{G}\left(\vec{r}\right)\right)\cdot g_{r_{j}}\left(\vec{r}\right)$ on $\vec{r}$ will be determined by the spatial distributions of the lattice deformation $\vec{u}\left(\vec{r}\right)$ and of the reciprocal lattice vector $\vec{G}\left(\vec{r}\right)$ throughout the sample.

### Sample Fabrication

The investigated samples were based on a 200‐nm‐thick GaAs_1‐_ *_x_* N*_x_* (*x* = 0.6%) epilayer, pseudomorphically grown by molecular beam epitaxy (growth temperature = 500 °C) on top of a 500‐nm‐thick, undoped GaAs buffer deposited on a (001) GaAs substrate. The surface of the epilayer was covered with ordered arrays of hydrogen silsesquioxane (HSQ) circular masks, defined by electron beam lithography (EBL, see Figure 2). The masked sample was then irradiated (at a temperature *T_H_* = 300 °C) with a low‐energy (100 eV) beam of H^+^ ions. The H dose *d_H_* = 10^18^ ions cm^−2^ was employed, corresponding to the exact amount of hydrogen required to ensure the full passivation of an unmasked GaAsN epilayer, as determined through a prior calibration run.

It was reminded to the reader that H forms at least two (if not more; see ref. [51]) stable N‐H complexes in GaAsN, with the so‐called N‐3H—which resulted in a lattice parameter exceeding that of GaAs^[^ ^46^ ^]^—being considerably more effective at deforming the GaAsN crystal than the N‐2H. Indeed, the latter merely neutralized the N‐induced reduction of the lattice constant, leading to a near‐perfect matching with pristine GaAs. Therefore, the hydrogenation conditions were optimized to establish the highest possible concentration of the N‐3H species within the sample, thus obtaining a maximally deformed lattice. As demonstrated by the concentration maps displayed in the lower two panels of Figure 2d—relative to the N‐2H and N‐3H complexes, respectively—the hydrogenated regions of the samples investigated here are nearly saturated with N‐3H complexes, i.e., the local concentration of the latter, *x_N‐3H_*, is basically identical to the initial N content (*x_N‐3H_* ∼ *x* = 0.6%). The N‐2H species is subsequently almost absent, but for a rather narrow region that marks the interface between fully hydrogenated and H‐free sections (as shown in the central panel of Figure 2d).

### FEM Calculations

The anisotropic H distribution inside the selectively hydrogenated GaAsN/GaAsN:H samples was computed by FEM calculations, performed according to the procedure described in ref. [53] and based on the set of diffusion‐reaction equations introduced in ref. [39] A 3D sketch of the computational domain is shown in Figure 2b: periodic boundary conditions were employed in the *xy* plane, wherein the domain was centered on a H‐opaque mask and had a lateral size equal to *w+a* (as indicated by the white‐dashed square displayed in Figure 2a). In the *z* direction, the upper surface was exposed to a constant flux of H^+^ ions (chosen to ensure that the computed hydrogenation time would match the experimental one), whereas continuous boundary conditions were applied at the bottom of the GaAs slab placed underneath the GaAsN epilayer (see Figure 2b).

As also discussed in ref. [53], the availability of the H distribution maps obtained in the previous step (see Figure 2c,d) is the starting point for a second set of FEM calculations, aimed at computing the effects of the different N‐H complexes (and of N) on the deformation of the surrounding lattice (see Figure 2e–j). Notably, this additional set of calculations was characterized by a crucial methodological detail, which is worth describing here. In the pseudomorphic growth regime, the in‐plane lattice constant of the deposited layers is expected to match that of the underlying substrate. Computationally, this is usually reproduced by placing a fixed constraint in the plane underneath the investigated structure. In the present case, however, it was taken into account that the H‐induced deformation of the GaAsN epilayer could trigger a distortion of the GaAs substrate in proximity of the interface, in a way akin to that reported in, e.g., ref. [54]. In order to accomplish this goal, a 1.5 µm‐thick GaAs slab was added in between the hydrogenated GaAsN layer and the fixed constraint (in the *xy* plane, periodic boundary conditions were employed, similar to those used for the simulations of the hydrogenation process). As shown in Figure 2g,h, the FEM calculations performed under these constraints do indeed suggest the presence of a small but non‐negligible lattice deformation in the upper portion of the GaAs substrate, underneath the selectively hydrogenated GaAsN layer.

### X‐Ray Transmission Measurements

The transmission of X‐ray photons through the crystal was monitored by scanning the angle *θ* between the incident beam—whose width was set to 50 µm—and the sample surface, rotating the sample in the *xz* plane (see Figures 1a,b and 3a–c). The source of the incident X‐rays was the XRD1 beamline of the Elettra Synchrotron in Trieste (Italy), whilst the sample was mounted on a kappa diffractometer equipped with a motorized goniometric *X–Y* stage head. The transmitted photons were collected by a water‐cooled Photonic Science X‐Ray Hystar 2048 CCD (pixel size of 3.8 µm), placed 80 cm downstream from the sample. To reduce absorption, prior to the measurements, the samples were mechanically thinned down to ≈100 µm. Also to minimize absorption by the sample, as well as by air, the X‐ray energy—selected by a double‐crystal Si(111) monochromator—was set to 10.33 KeV, just below the K‐edge of Ga. Given that—according to the theoretical background laid out in the dedicated Subsection of the Methods—the Berry‐phase translation effect is expected to be maximal when the system fulfills the Bragg condition, the investigated angular range was centered on *θ*_B_ = 25.12°, the Bragg angle corresponding to the reciprocal lattice vector relative to the (004) reflection of GaAs.

It is worth noting here that the configuration employed for these measurements were actually nearly identical to the one employed in conventional X‐ray rocking curve (XRC) experiments: aside from the obvious differences—in XRC one measures the beam diffracted by the sample's surface (characterized by the wavevector ${\vec{k}}^{\prime }$, see Figure 1a), rather than the X‐ray photons transmitted through the crystal—for both measurement techniques the photon wavelength *λ* is kept constant (so that $k=\frac{2\pi }{\lambda }$ also remains fixed) and $\vec{k}$ is varied by changing *θ*. Before each set of transmission measurements, indeed, the sample orientation corresponding to the correct value of the *θ*_B_ angle was identified by maximizing the diffracted signal collected by a second CCD camera placed in theta/2‐theta configuration with respect to the sample and to the incoming beam, which basically amounts to performing a quick XRC scan.

Based on the expectations of the theoretical model outlined above—and introduced in ref. [19, 20, 21]—when *θ* is either much smaller or much larger than *θ*_B_, the path of the transmitted photons should be virtually unaffected by the presence of the sample, and the CCD camera should detect a single bright spot, collinear to the incident beam (see Figure 3a). When *θ* is in close proximity to *θ*_B_, on the other hand, the transmitted X‐rays should display the large translations associated with the Berry‐phase effect, and multiple, distinct features (associated with different regions of our artificially structured samples) should appear on the CCD (see Figure 3b,c). Notably, the sketches reported in Figure 3a–c are drawn in such a way as to be consistent with an experimentally verified feature of the transmitted X‐ray photons, i.e., with the fact that all the translated beams are entirely contained in the plane (labeled as *xz* in the figure) defined by the incident beam and by $\;{\vec{G}}_{0}$ (which is, in turn, parallel to the *z* axis and perpendicular to the sample surface). Because of this peculiar property—which is theoretically explained in Note S3 (Supporting Information)—all the translated beams impinge on the CCD along a clearly defined line (parallel to the *x*_CCD_ axis, according to the notation specified in Figure 3a–c), and the Berry‐phase effect can be fully characterized in terms of the beam translation along this line.

## Conflict of Interest

The authors declare no conflict of interest.

## Supporting information



Supporting Information

## Data Availability

The data that support the findings of this study are available from the corresponding author upon reasonable request.
