# Supplementary material for: Giant Berry‐phase‐Driven X‐Ray Beam Translations in Strain‐Engineered Semiconductor Crystals
Source: Adv Mater. 2025 Oct 30;38(11):e13259. doi: 10.1002/adma.202513259 (PMC12921357; doi:10.1002/adma.202513259)
Supplement: Supplementary file 1 — Supporting Information [file ADMA-38-e13259-s001.docx]

Supporting Information

Giant Berry-phase-driven X-ray beam translations in strain-engineered semiconductor crystals

Marco Felici*, Giorgio Pettinari, Michela Fratini, Luisa Barba, Simone Birindelli, Gaetano Campi, Silvia Rubini, Tobias Schülli, Mario Capizzi, and Antonio Polimeni

**Contents:**

**Supplementary Note 1. Reciprocal lattice vectors in a deformed lattice** Page 2

**Supplementary Note 2. Analytic relationship between
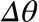
,
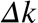
, and *w*_Berry_** Page 4

**Supplementary Note 3. Modeling of the Berry-phase effect in strain-engineered GaAsN:H** Page 9

**Supplementary Note 4. Visibility range of the Berry-phase effect** Page 13

**Supplementary Figure S1.** **Modeling of the Berry-phase effect in strain-engineered GaAsN:H.** Page 15

**Supplementary Figure S2.** **Relationship between
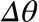
,
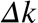
,
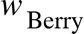
, and
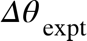
.** Page 17

(**NOTE**: The references cited in the Supporting information follow the same numeration of those in the main manuscript.)

**Supplementary Note 1. Reciprocal lattice vectors in a deformed lattice**

In the following, we provide an explicit expression of the position-dependent reciprocal lattice vector,
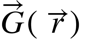
, in a deformed crystal (sketched in Figure 1c of the main text). Throughout this work, such vector is identified with the one relative to the (004) reflection, equal to
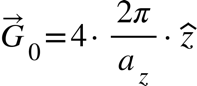
 in undeformed GaAs (see Figure 1a of the main text) and relevant to the employed experimental configuration (see Figure 3). Provided that the lattice deformation occurs over length scales much larger than the lattice constant—*i.e*., that the crystal retains its periodicity at the nanometric level, so that a reciprocal lattice can still be defined— in a deformed crystal the
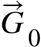
 vector can be rewritten as


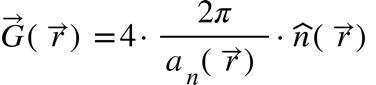
, (S1)

where
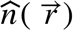
 is the versor normal to the surface defined by the distorted (001) lattice plane containing
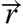
 and
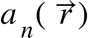
 is the lattice constant in the direction parallel to
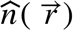
.

In order to numerically estimate
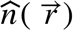
, we must consider that the outputs of all the FEM calculations described in the manuscript are sampled on a regular three-dimensional grid, made up of all the points identified by the equation


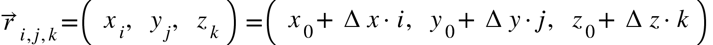
, with
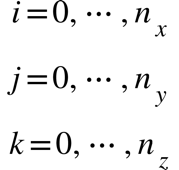
. (S2)

Here,
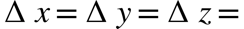
10 nm, while
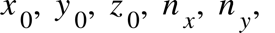
 and
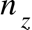
 are chosen to yield
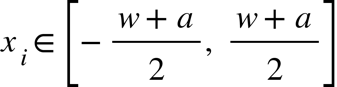
,
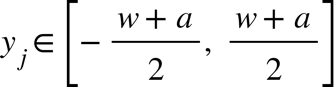
, and
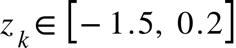
, thus spanning the whole computational domain (see Figure 2b in the main text). If
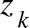
 is kept constant, *i.e*., if we fix
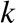
 to an arbitrary value (*e.g*.,
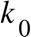
), all the points that respect the condition


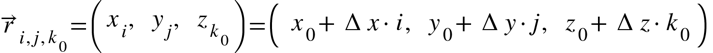
 (S3)

fall on a plane, perpendicular to the [001] crystallographic direction. If, in turn, each one of these points is translated by its corresponding lattice deformation vector,
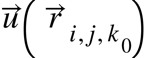
, we obtain


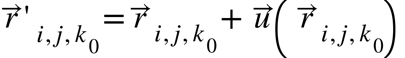
. (S4)

Put together, the
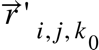
 points numerically define a deformed surface, whose normal vector
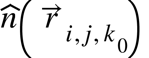
 can be easily computed. By repeating this procedure for all possible values of
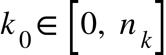
, it is then rather straightforward to obtain the
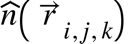
 vector in every point of our computational domain. Since the
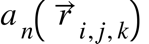
 lattice vector in the
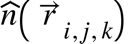
 direction actually coincides with the computed lattice vector in the *z* direction,
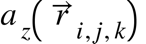
—the way the *z* axis is defined in the calculations is based on the regular grid made up of the
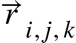
 points, *i.e*., it does not explicitly account for the lattice distortion—we can, thus, directly estimate
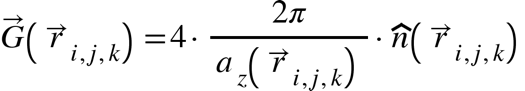

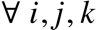
 (see Figure 2i-j in the main text).

**Supplementary Note 2. Analytic relationship between
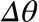
,
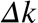
, and *w*_Berry_**

As noted in the main text,
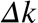
 is the width of the gap forming in the dispersion relation of X-ray photons due to the crystal’s periodicity, *i.e*., the extent of the interval for which a perfect crystal optimally diffracts/reflects the incoming beam, so that its propagation in the lattice is strongly inhibited. In X-ray diffraction, on the other hand, the Darwin width
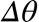
 is defined as the angular range for which the intensity of the diffracted beam—measured as a function of the angle *θ* between
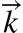
 and the sample surface (see, *e.g*., Figure 1a-b of the main text)—is maximal.^[12]^ Given these definitions, it is relatively straightforward to establish a simple geometric relationship between
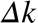
 and
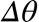
 (as sketched in Figure 1b). If we define
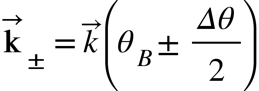
, we can indeed write


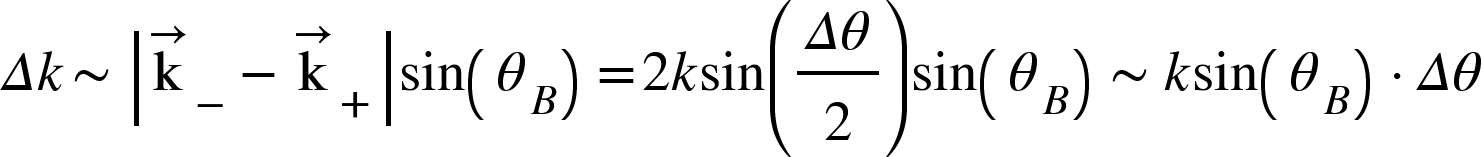
, (S5)

from which it follows immediately that


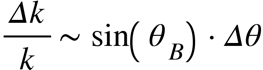
. (S6)

The comparison between this equation and Equation (5) in the Methods section of the main manuscript immediately yields


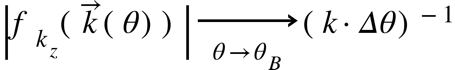
, (S7)

thus allowing us to establish a direct relationship between the maximum of
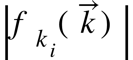
 and a quantity that can be measured directly, such as
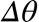
. For the experiments discussed in the main text, the incident beam was swept around the Bragg angle corresponding to the (004) reflection of GaAs, which for the beam energy we employed (10.33 keV) is equal to *θ_B_*=25.12°. Under similar conditions, the Darwin width for a perfect GaAs crystal was theoretically estimated^[65]^ to be
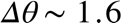
 mdeg, or
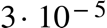
 rad, leading to
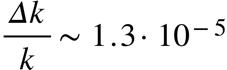
. Not only is this value perfectly consistent with those reported in Reference [19–21] (and leading to the macroscopic beam translations observed therein); it is also in good qualitative agreement with the value providing the best match between our model and the experimental data, equal to
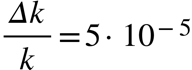
 (see main manuscript).

Having just established a direct relationship between
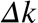
 and
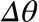
 [see Equation (S6)], in the following we investigate the evolution of
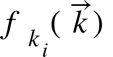
—and, thus, of the Berry-phase effect—as a function of the value taken by
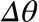
 in a given system. For the reader’s convenience, we begin this section by rewriting Equation (3) of the main manuscript, wherein the
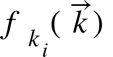
 term was introduced:


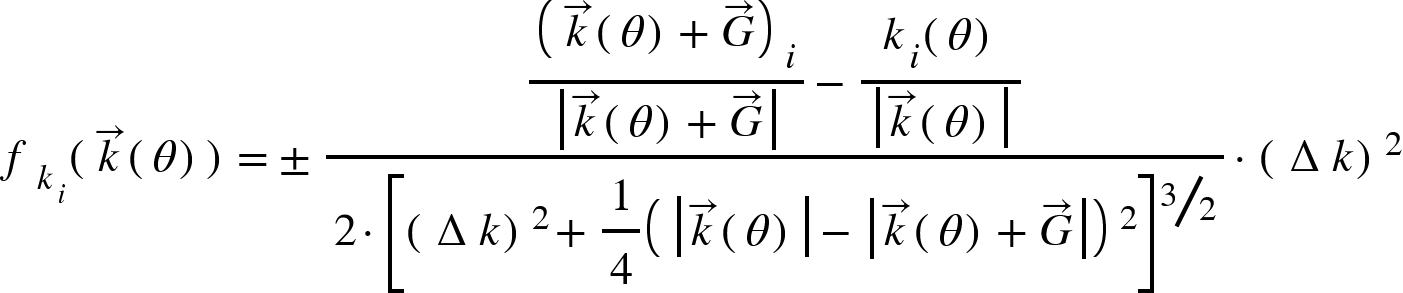
. (S8)

In our experiments the photon wavelength, *λ*, was kept fixed, so that
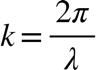
 also remained constant, and
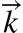
 was changed solely by varying the angle *θ* between the incident X-ray beam and the sample surface (see Figure 1 of the main text). As a result, the dependence of
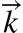
 on *θ* can be made explicit, *i.e.*,


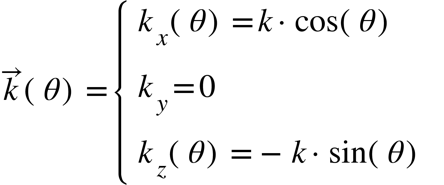
. (S9)

Furthermore, as sketched in Figure 1c-e of the main text, in an arbitrarily deformed medium
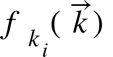
 is also a function of the position within the lattice,
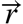
, due to the spatial dependence of the reciprocal lattice vector,
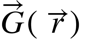
. Based on these considerations, we can redefine
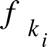
 as
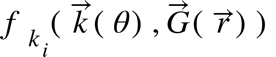
, which, in turn, can be rewritten as
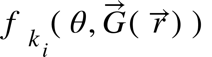
. For the purposes of the present discussion, however, we can fix
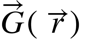
 to
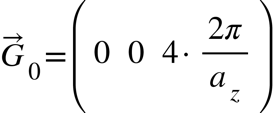
, the reciprocal lattice vector associated with the (004) reflection of undeformed GaAs (see Figure 1 of the main text). Without leading to any loss of generality, indeed, this choice ensures a series of simplifications, chiefly associated with the fact that
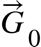
 is parallel to the *z* axis. As also discussed in the main text [see Equation (5)], this entails that
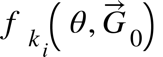
 is nonzero only for *k_i_* = *k_z_*, with the absolute value of
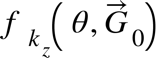
 being equal to


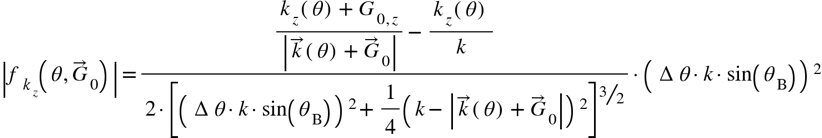
. (S10)

Given that—according to the Bragg condition—
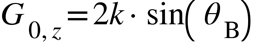
, and that
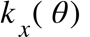
and
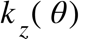
 are defined in Equation (S9), we can also write


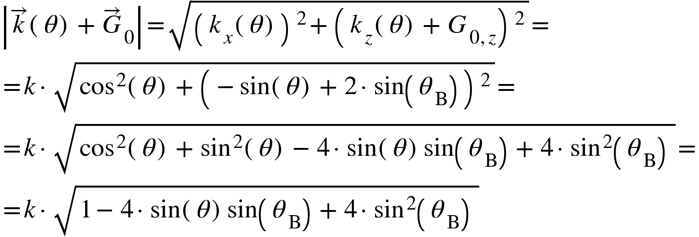
. (S11)

If we put together Equation (S9), (S10), and (S11), we obtain


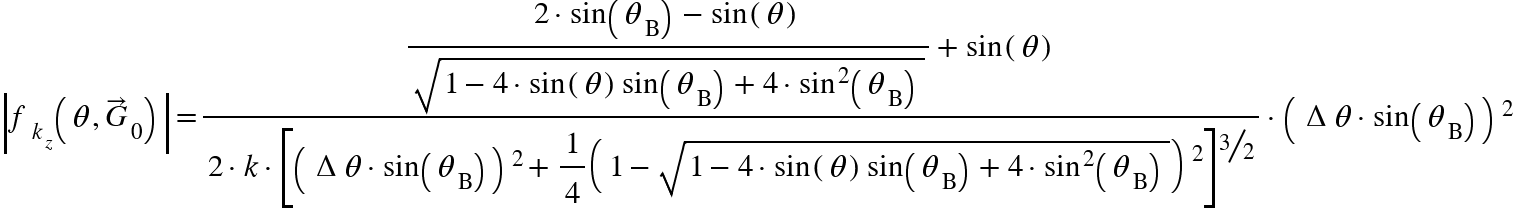
. (S12)

As shown in the inset of Supplementary Figure S2a,
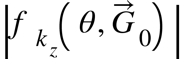
 is a function peaked at the Bragg angle, *θ*_B_, with
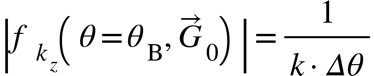
 [see Equation (S7)]. As discussed in Supplementary Note 4, the full width at half maximum (FWHM) of such function, *w*_Berry_, is also directly related to the angular range over which the Berry-phase effect can be experimentally observed. It can thus be useful to investigate the analytic relationship between *w*_Berry_ and the Darwin width
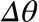
, which, apart from its well-known physical significance (see main text), also features as an important parameter in Equation (S12).

According to the definition of FWHM, *w*_Berry_ should respect the condition


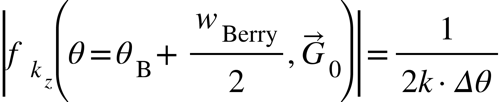
. (S13)

Before we can apply this condition to the situation at hand, however, it is useful to note that


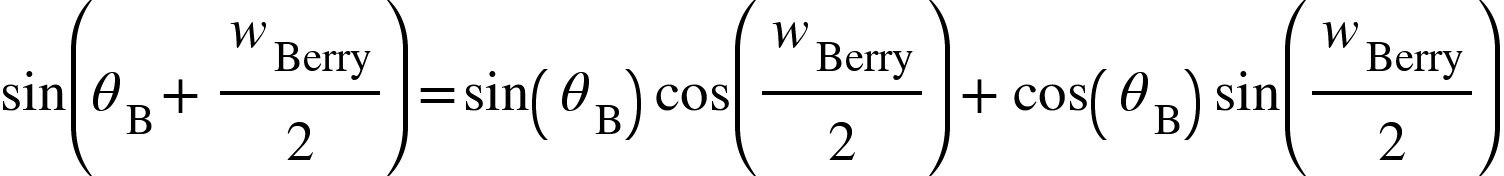
, (S14)

which, given that typical *w*_Berry_ values are in the 10-100 mdeg (i.e., ~2⋅10^-4^-2⋅10^-3^ rad) range (see Supplementary Figure S2a), can be approximated to


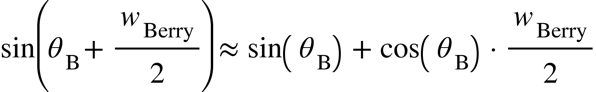
. (S15)

In addition, it is also useful to note that


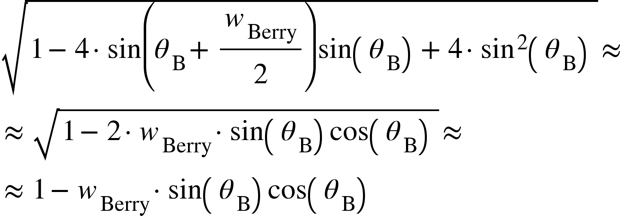
. (S16)

If we plug Equation (S15) and (S16) into Equation (S12), we obtain


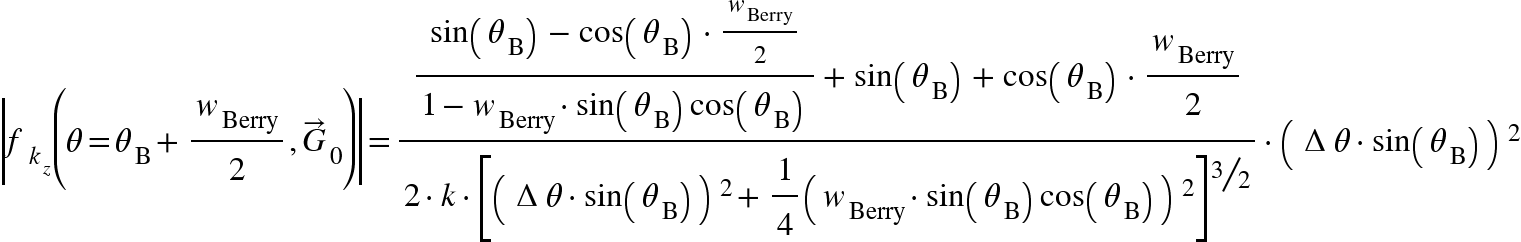
, (S17)

which can be simplified to


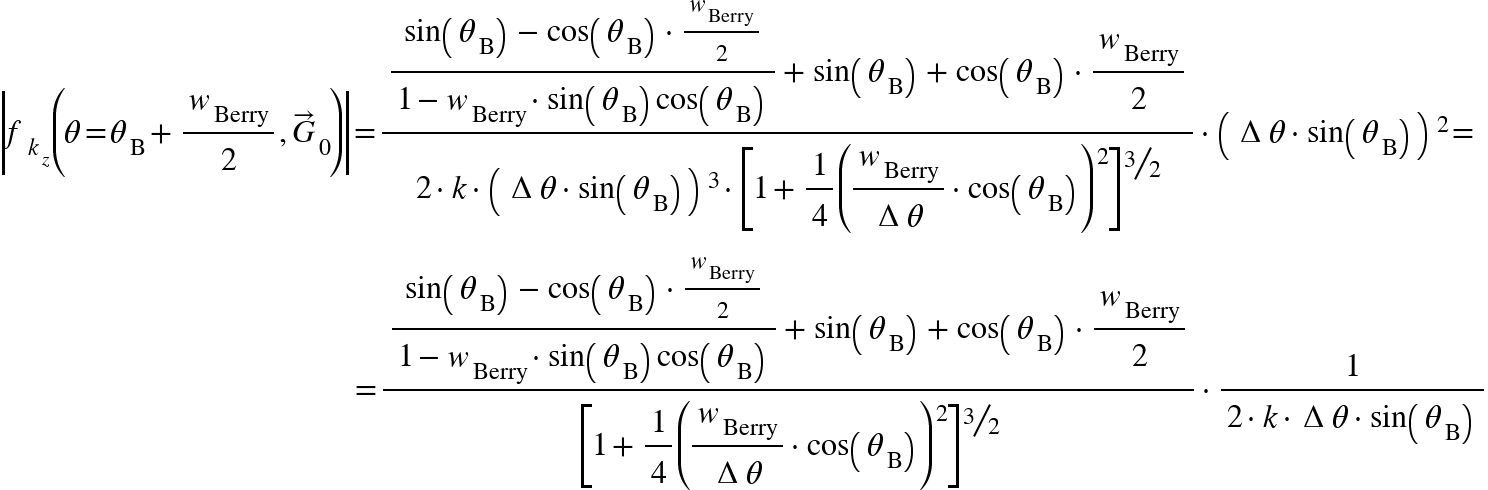
. (S18)

In turn, this expression can be approximated to


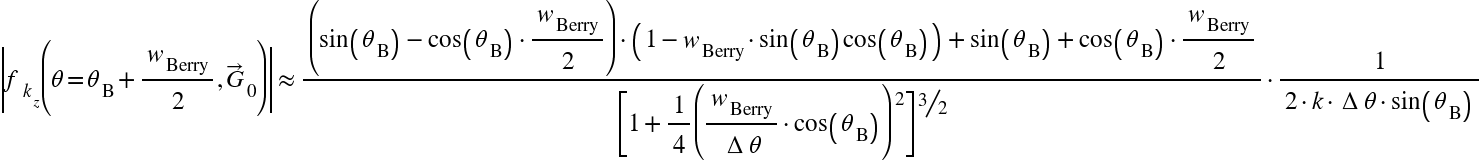
, (S19)

which then becomes


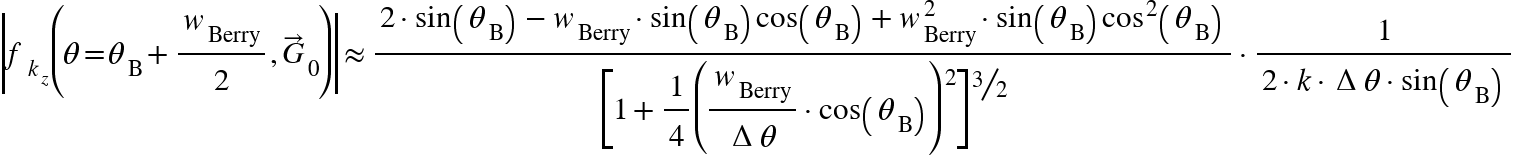
. (S20)

At this point, we can further approximate this expression, by eliminating all the terms containing *w*_Berry_ from the numerator (at the denominator, *w*_Berry_ only appears in a ratio with
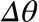
, and since
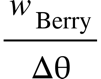
 ~ 1 it cannot be eliminated):


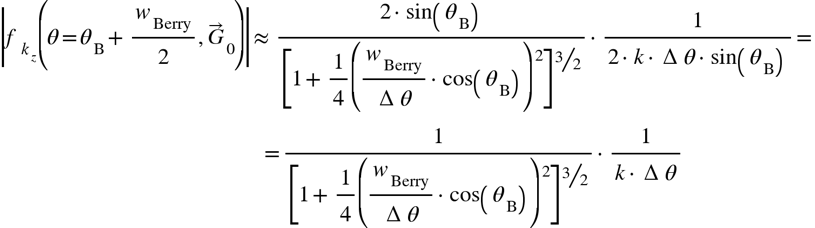
. (S21)

Finally, if we apply the condition defined in Equation (S13) to (S21), we obtain


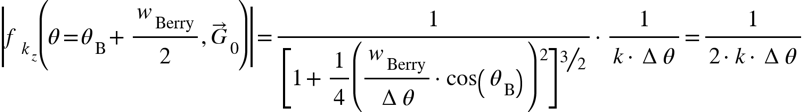
, (S22)

which yields


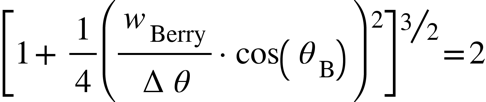
 (S23)

and, finally,


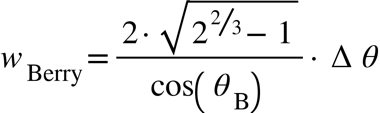
. (S24)

As shown in the main panel of Supplementary Figure S2a, the dependence of *w*_Berry_ on —as estimated by numerically computing for several values of —is in near-perfect agreement with Equation (S24), thus confirming the existence—within the approximations made above—of a linear relationship between *w*_Berry_ and .

**Supplementary Note 3. Modeling of the Berry-phase effect in strain-engineered GaAsN:H**

As noted in the main text, the availability of the complete spatial distribution maps of and within the investigated samples allows us to estimate , which can then be plugged into Equation (1) of the main manuscript to gain insight into the propagation of X-rays in our system. In order to achieve this goal, we must first of all express the aforementioned Equation (1) as

. (S25)

In turn, this equation can be rewritten as

, (S26)

or, more explicitly,

. (S27)

This system of equations can be greatly simplified if we take into account the specific properties of our system (and of our experimental configuration). First of all, the vector of the impinging X-ray beam lies entirely in the *xz* plane (see Figure 1a-b), so that . Furthermore, as noted in Supplementary Note 1, the components of in the *xy* plane—albeit very important for the determination of —are much smaller than its component in the *z* direction (*e.g.*, for the example displayed in Figure 2i-j), so that it is always possible to assume [see Equation (5), in the Methods section of the manuscript]. In light of these considerations, Equation (S27) can be approximated to

(S28)

or, if the sum over is written out in full, to

. (S29)

These three equations can now be merged, resulting in the expression

, (S30)

which finally yields

. (S31)

If we subtract [= ] to this quantity, and then we normalize to , we obtain

, (S32)

which can be rewritten as

(S33)

and represents the relative change—with respect to a straight beam—of the *z* component of the equation of motion for an X-ray wave packet propagating in a deformed medium. This obviously entails that, for the experimental configuration employed in this work (see, *e.g*., Figure 3), the Berry-phase effect adds an extra term to the *z* component of the group velocity of the propagating X-ray photons, leading to a change of the beam trajectory inside the medium and ultimately resulting in the net translations measured for the emerging X-rays. We do indeed have a *translation*—*i.e*., the transmitted X-ray beams are parallel to the incident one—rather than a (perhaps more trivial) beam deviation, because the Berry phase-related term goes to zero as soon as the X-rays exit the deformed crystal, yielding a recovery of the initial propagation direction. In addition, the fact that the crystal deformation only affects the motion of the X-ray photons in the *z* direction clearly implies that the transmitted X-rays must continue to lie in the *xz* plane, in agreement with the considerations we made when discussing Figure 3 and 4.

If we now return to our analysis of , we must note that the latter was defined, in Equation (S33), as a function of the position inside the sample, , and of the wavevector of the incident X-ray beam, . As already mentioned in the main manuscript, however, throughout our experiments was varied by simply changing the angle *θ* between the incident X-rays and the sample surface, so that we can rewrite . The possibility of using Equation (S33) to evaluate for any given angle *θ* and in every point of the computational domain (see, *e.g*., Figure 3a-b) provides us with the ability to estimate the dependence on *θ* of the *distribution* of the values taken by within our system, which we will label as . As we will see in the main text, this quantity can be directly—albeit qualitatively—compared with our experimental results, greatly aiding our interpretation of the latter. In order to numerically construct , we proceed as follows: for a fixed angle, , we build a histogram with all the values taken by within the computational domain, thus obtaining a good numerical approximation of , the distribution of for the angle . Then, this procedure is repeated for a set of different angles (, , …), covering the angular range of interest. The series of one-dimensional functions that result from this procedure [, with ] can be stacked together to form a two-dimensional array, which represents a good numerical approximation of the function.

In panel a of Supplementary Figure S1, the full distribution relative to the sample with *a* = *w* = 1 µm is plotted—in a logarithmic scale—as a function of *θ* – *θ*_B_, over an angular range large enough to encompass all the values taken by across the sample (see Figure 3e). is reported in units of *v_g_*, the group velocity of X-rays undergoing “conventional” transmission (*i.e.*, propagating in the medium without being affected by the Berry-phase effect). Quite clearly, presents several distinct features, with markedly discernible traces characterized by a well-defined dependence on *θ* – *θ*_B_, and by maximal values of that exceed *v_g_* by nearly one order of magnitude. In order to shed light on the microscopic origin of these features, however, it becomes necessary to separate the contributions of the different regions of the sample to . This is done in Supplementary Figure S1b-d, which refer, respectively, to the distributions of relative to the hydrogenated (Panel b) and untreated (Panel c) portions of the GaAsN epilayer, and to the GaAs substrate (Panel d). As these maps clearly show, the features appearing at an angle *θ* < *θ*_B_ overwhelmingly originate from the hydrogenated regions, consistent with the fact that, on average, is sizably smaller than *θ*_B_ in these areas (see Figure 3d). For the untreated sections of the sample, on the other hand, is generally larger than *θ*_B_, and the contribution of these regions to is accordingly predominant for *θ* > *θ*_B_. Finally, the features displayed in Supplementary Figure S1d—mostly concentrated around *θ* ~ *θ*_B_—stem from the local deformations of the GaAs substrate, clearly visible in, *e.g.*, Figure 2g-h of the main manuscript and triggered by the H-induced distortion of the surmounting GaAsN epilayer.

While Supplementary Figure S1a-d display over an extended angular range (~400 mdeg), comprising the entire distribution of within the sample, from the experiments we know—as discussed in the manuscript and in Supplementary Note 4 (see also Supplementary Figure S2b)—that the Berry-phase effect is only visible over a considerably smaller interval, centered on *θ*_B_ and having width ~ 20-40 mdeg. Panel e of Supplementary Figure S1 presents a close-up of over a ~ 60 mdeg range, identical to the one characterizing the measured transmission patterns featured in Figure 4a-c (see also Supplementary Note 4 and Supplementary Figure S2b). Panels f-h, on the other hand, single out—in a way akin to Panels b-d—the contributions of the hydrogenated (f) and untreated (g) sections of the GaAsN epilayer, as well as those of the underlying GaAs substrate (h). As briefly noted above, the comparison between and the experimental data—boosted by our ability to discern the importance of the role played by each region of the sample, see Supplementary Figure S1f-h—will greatly aid our understanding of the dependence of the Berry-phase translation effect on the geometry of the deformation pattern (as discussed in the dedicated section of the main manuscript).

**Supplementary Note 4. Visibility range of the Berry-phase effect**

Finally, it is worth mentioning that the extent of the angular range over which the Berry-phase effect is experimentally observable is not easy to predict, due to a series of sample-dependent factors. First of all, as already noted in the main manuscript, in a deformed crystal the reciprocal lattice vector relative to the (004) reflection, , is position-dependent, and so is the associated Bragg angle, . If the effects of the modulation of the lattice parameter across the sample are paired with those of the tilt of the orientation of in the deformed crystal, the extent of the fluctuations of within the lattice is expected to be of the order of ~100-200 mdeg. These expectations are confirmed by Figure 3e, which displays the distribution of for the samples investigated here (the effective N concentration for these same samples is displayed in Figure 3d).

In addition, as also discussed above, in any given point of the lattice the dependence of the Berry-phase effect on —and, thus, on *θ*—is entirely contained within the function [see Equation (S8)] whose absolute value is a curve peaked at (see the inset of Supplementary Figure S2a for an example). In Supplementary Note 2, the full width at half-maximum of this curve—labeled as *w*_Berry_—was analytically demonstrated to be directly proportional to the Darwin width of the crystal, [see Equation (S24)], and thus, if Equation (S6) is properly taken into account, to the value of the parameter:

. (S34)

As noted in the main text, the best agreement between our model and the experiments is achieved for , which—when inserted in Equation (S34)—yields = 11 mdeg.

In principle, one would expect the angular range of the Berry-phase effect to result from the convolution of with the distribution of across the sample. Given that —our best estimate for the width of for the system investigated here—is much smaller than the variability of within our samples (11 mdeg vs. 100-200 mdeg, see above and Figure 3e of the main manuscript), it would be reasonable to expect the latter to dictate the extent of the “visibility range” of the Berry-phase effect, which should thus be of the order of a few hundreds of mdeg.

Looking at the experimental data reported in Figure 4 of the main manuscript, however, it is quite clear that the visibility interval of the Berry-phase effect is considerably narrower. As a matter of fact, if Figure 4 is compared with Supplementary Figure S2b—which presents the measured angular dependence of the integrated intensity of the signal transmitted through the samples—one can easily realize that the Berry phase effect is only visible within the low-intensity window present in the integrated transmitted signal, whose width, , ranges between 21 and 35 mdeg for the samples investigated here. This low-transmission region, centered around *θ*_B_ for all samples, corresponds to the angular window within which the incident X-rays are ~100% diffracted by the crystal—or, to be more precise, by the GaAs substrate (*θ*_B_ is the Bragg angle corresponding to the reciprocal lattice vector of GaAs, as discussed above). It is only in this narrow region, wherein conventional transmission is theoretically forbidden (see also Figure 1), that it becomes possible to observe the comparatively weak signal associated with “anomalously” transmitted photons, *i.e*., those subjected to the Berry-phase effect itself. It is, also, hardly surprising to note that is systematically *larger*—albeit of the same order of magnitude—than = 6.7 mdeg, the Darwin width that results from plugging into Equation (S6). Indeed, while should approximately correspond to the Darwin width of a homogeneous, undeformed, “GaAs-like” crystal, the value of is expected to incorporate the broadening effects of the large strain and composition gradients present in our patterned GaAsN:H/GaAs samples.

**Supplementary Figure S1.** **Modeling of the Berry-phase effect in strain-engineered GaAsN:H.** (a) (Main) False-color plot of the base-10 logarithm of , the dependence on of the distribution of within the deformed lattice (see Supplementary Note 3). is in units of *v_g_*, the group velocity of the X-ray wave packet in an undeformed crystal. The displayed plot refers to the sample with *a = w* = 1 µm, while the value of the parameter was set to . This value maximizes the model’s agreement with the experimental data (see main text) and was thus kept constant throughout the calculations displayed in the remainder of this work. (Inset) Section of the sample in the *y* = 0 plane, highlighting the untreated (blue) and hydrogenated (red) regions of the GaAsN epilayer, as well as the GaAs substrate (green). (b-d) Same as (a), but singling out the contributions to of the three sample regions identified in the inset of Panel (a). Panels (b) and (c) refer to the hydrogenated barriers and to the untreated regions, respectively, while (d) shows the contribution of the substrate to [the color scales employed for the different regions are consistent with the scheme introduced in the inset of Panel (a)]. (e) Zoom-in of in the same angular range of the experimental transmission intensity profiles displayed in Figure 4 of the main manuscript. The mapping of the color scale employed in this panel (and in the following ones) is linear—contrary to Panels (a-d), wherein it was logarithmic. Also, the scale is artificially saturated to enhance the visibility of the finer features of in the displayed range. The region shaded in dark yellow corresponds to = 11 mdeg, see above). The range corresponding to , the width of the low-intensity region experimentally observed in the integrated transmission signal (see Methods), is also highlighted in light yellow. (f-h) Same as (e), but separately showing the contributions to of the three sample regions identified in the inset of Panel (a): the plots relative to the hydrogenated and untreated regions are displayed in (f) and (g), whereas (h) refers to the GaAs substrate.

**Supplementary Figure S2.** **Relationship between , , , and .** (a) (Main) Dependence of *w*_Berry_ on (bottom horizontal axis) and (top horizontal axis). The “numerical” values of *w*_Berry_, reported as black dots, were obtained by graphically estimating the FWHM of the dependence of the function on , numerically computed for several different values of (see Supplementary Note 2). The expected linear dependence of *w*_Berry_ on [see Equation (S24), in Supplementary Note 2] is displayed as a red line. The gold star corresponds to = 11 mdeg, which results from the value of providing the best agreement between FEM calculations and experiments ( = ). (Inset) Dependence of the function on , numerically computed for = 10 mdeg. (b) Integrated intensity (see Supplementary Note 4) of the X-rays transmitted through the samples patterned with an array of circular masks having spacing *a* = 1 µm and mask size *w* = 0.2 µm (bottom, gray), 0.5 µm (center, red), and 1 µm (top, blue). For each sample, transmission was measured as a function of the difference between *θ*, the angle between the incoming beam and the sample surface, and *θ*_B_, the Bragg angle corresponding to the reciprocal lattice vector of GaAs (*θ*_B_=25.12°, see main text). For each sample we report the measured value of , the width of the low-transmission region, wherein X-rays are primarily diffracted by the sample (see Supplementary Note 2).
